# Combating Gram-negative infections: The role of antimicrobial peptides and nanotechnology in overcoming antibiotic resistance

**DOI:** 10.1016/j.mtbio.2025.102381

**Published:** 2025-10-04

**Authors:** Christian S. Carnero Canales, Jessica Ingrid Marquez Cazorla, Renzo Marianito Marquez Cazorla, Rafael Miguel Sábio, Hélder A. Santos, Fernando Rogério Pavan

**Affiliations:** a Vicerrectorado de Investigación, Universidad Autónoma del Perú (UA), Lima, 150142, Republic of Peru; b School of Pharmacy, Biochemistry and Biotechnology, Santa Maria Catholic University (UCSM), Arequipa, 04013, Republic of Peru; c Nanovida Research Center, Arequipa, 04451, Republic of Peru; d Tuberculosis Research Laboratory, School of Pharmaceutical Sciences, São Paulo State University, Araraquara, 14800-903, Brazil; e Department of Biomaterials and Biomedical Technology, The Personalized Medicine Research Institute (PRECISION), University Medical Center Groningen, University of Groningen, Groningen, 9713 AV, the Netherlands

**Keywords:** Gram-negative bacteria, Antimicrobial peptides, Nanotechnology, Nanocarriers, Drug delivery systems

## Abstract

Gram-negative bacterial infections represent a critical global health threat due to rising antibiotic resistance, posing substantial clinical and economic burdens. The structural complexity of Gram-negative bacteria—including efflux pumps, enzymatic degradation mechanisms, genetic mutations, and altered membrane permeability, significantly complicates treatment with conventional antibiotics. Antimicrobial peptides (AMPs) offer great antimicrobial activity, rapid bactericidal effects, multiple mechanisms of action, and immune-modulatory properties. However, their clinical application is limited by enzymatic degradation, potential cytotoxicity, and high production costs. To address these limitations, advanced nanotechnology-based platforms, including lipid-based nanoparticles, polymeric nanoparticles, and inorganic nanoparticles, are being explored as AMP delivery vehicles. These nanocarriers enhance peptide stability, bioavailability, targeted delivery, and therapeutic efficacy while reducing systemic toxicity. This review synthesizes recent advancements in AMPs and nanotechnology-based delivery systems, highlighting their combined potential to effectively combat multidrug-resistant Gram-negative pathogens.

## Graphical abstract

**Figure:**
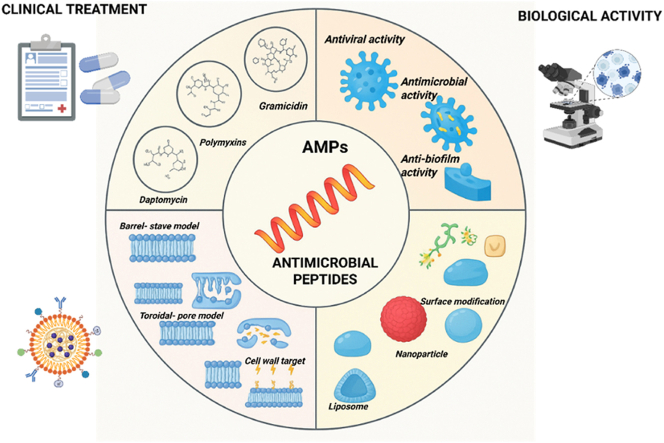

## 1 Introduction

The emergence and global spread of multidrug-resistant (MDR) Gram-negative bacteria represent one of the most critical challenges in modern medicine [1]. These pathogens are responsible for a wide range of nosocomial and community-acquired infections and are characterized by their intrinsic and acquired resistance to multiple classes of antibiotics [2]. Unlike Gram-positive organisms, Gram-negative bacteria possess a highly complex cell envelope that includes an outer membrane rich in lipopolysaccharides, contributing to reduced membrane permeability and enhanced defense against antimicrobial agents [3]. In addition, they employ diverse resistance mechanisms, including the production of inactivating enzymes such as β lactamases, alterations of target sites, decreased antibiotic uptake through porin loss, and the coordinated action of efflux pumps, all of which collectively hinder the effective treatment of infections [4,5].

High priority Gram-negative pathogens such as *Acinetobacter baumannii*, *Pseudomonas aeruginosa* and carbapenem resistant Enterobacterales (CRE) are recognized by the World Health Organization (WHO) as urgent threats due to their increasing prevalence and the scarcity of effective therapies [6]. These organisms, exemplified by carbapenem resistant *A. baumannii* (CRAB), carbapenem resistant *P. aeruginosa* (CRPA) and extended-spectrum β-lactamase (ESBL) producing or CRE [7], represent some of the most critical therapeutic barriers. In addition to their severe impact on patient outcomes, they impose a substantial economic burden on healthcare systems worldwide [8]. Although new agents such as cefiderocol, a siderophore cephalosporin, provide activity particularly against *P. aeruginosa* and *A. baumannii*, therapeutic options remain limited overall [9]. The rapid dissemination of resistance frequently undermines the efficacy of these drugs and often forces reliance on colistin, a last line antibiotic restricted by nephrotoxicity and high mortality risk [10]. Within this context, alternative therapeutic strategies have gained increasing relevance, with antimicrobial peptides (AMPs) standing out as promising candidates to address the limitations of conventional antibiotics. AMPs have attracted considerable attention as alternative therapeutic agents capable of overcoming traditional resistance mechanisms. These peptides act primarily by disrupting bacterial membranes and may also exert intracellular and immunomodulatory effects [11]. Their fast-acting nature and ability to bypass classical resistance pathways position them as strong candidates in the development of next-generation antimicrobials [12]. However, AMPs face critical limitations that hinder their clinical translation, including poor stability under physiological conditions, enzymatic degradation, cytotoxicity, and low bioavailability [13,14].

To address these challenges, nanocarrier-based delivery platforms have been developed to enhance the therapeutic performance of AMPs [15]. By encapsulating or conjugating AMPs with nanostructured materials—including lipid-based, polymeric, and inorganic systems—it is possible to achieve improved protection against proteolysis, controlled release profiles, enhanced bacterial targeting, and reduced systemic toxicity [16]. These multifunctional nanocarriers not only improve pharmacokinetics but also exhibit intrinsic properties that synergize with AMP activity, offering a powerful strategy to combat resistant Gram-negative infections [17].

This review integrates bacterial resistance mechanisms, peptide modes of action, and nanocarrier design, linking key barrier-level challenges (lipopolysaccharide traversal, efflux, biofilm penetration, intracellular reservoirs) to the selection of lipidic, polymeric, and inorganic nanoplatforms. In addition, we provide comparative tables that compile reports from the past five years on active peptides-loaded or conjugated nanoplatforms against major Gram-negative bacteria, including both antibiotic-sensitive and resistant strains, thereby offering a systematized and up-to-date overview of the field.

## 2 Bacterial resistance to conventional antibiotics

**Fig. 1:**
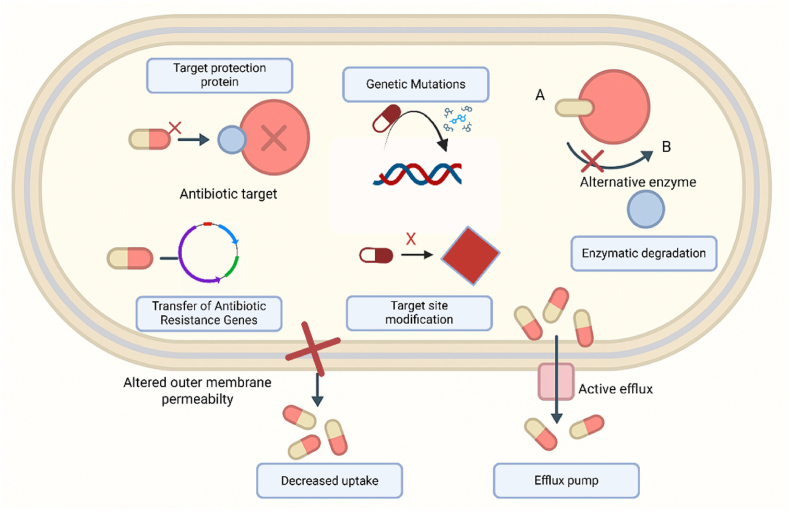
Representation of antibiotic resistance mechanisms in a Gram-negative bacterium, including enzymatic inactivation, target modification, gene transfer, decreased uptake, and active efflux.

Resistance among Gram-negative pathogens arises from multiple and overlapping mechanisms, including reduced outer-membrane permeability and porin alterations [18], active efflux mediated by resistance-nodulation-division (RND) pumps [19], enzymatic inactivation by β-lactamases [20], and horizontal gene transfer via plasmids, integrons, transposons, and phages (Fig. 1) [21].

According to the 2024 WHO priority list, CRAB, CRPA, and CRE/ESBL are among the most concerning threats [6,22]. These organisms are strongly associated with severe hospital-acquired infections such as pneumonia, septicemia, and urinary tract infections, and together account for the majority of Gram-negative isolates worldwide [20]. Their high capacity for dissemination and persistence in clinical settings, combined with their ability to rapidly acquire and share resistance determinants, has made them especially difficult to control [23]. The clinical burden is reflected in increased mortality, prolonged hospital stays, and escalating healthcare costs [24].

Although new agents such as cefiderocol have shown activity against some MDR strains, resistance frequently undermines their efficacy, often leaving colistin as the last therapeutic option despite its nephrotoxicity and limited effectiveness [9,10]. This scenario underscores the urgent need for therapeutic alternatives capable of overcoming multifactorial resistance. Within this framework, AMPs stand out for their multimodal mechanisms, rapid bactericidal activity, and reduced propensity to induce resistance [25].

## 3 Antimicrobial peptides

AMPs, also known as host defense peptides, are secondary metabolites produced by all living organisms and play a fundamental role in innate immunity [26]. These peptides are short polypeptide molecules (10–100 amino acids) with a broad spectrum of antimicrobial activity against bacteria (both Gram-positive and/or Gram-negative), viruses, fungi, and protozoa. Additionally, they exhibit diverse chemical properties and target cells, enabling them to act through multiple mechanisms and generate various physiological effects depending on their interactions with intracellular components and lipid membranes [27,28].

AMPs are considered promising therapeutic molecules due to their dual advantages: while they possess broad-spectrum activity, they can also be specifically designed to target pathogens. This adaptability makes them an attractive alternative to conventional antibiotics, particularly in the face of rising antimicrobial resistance [29]. Unlike traditional antibiotics that often act on a limited number of bacterial targets, AMPs function through multiple mechanisms, such as disrupting membrane integrity, interfering with intracellular processes, or modulating the host immune response [30,31]. Moreover, their rapid mode of action helps prevent the development of resistance by targeting conserved bacterial structures such as phospholipid layers or peptidoglycan, and AMPs make effective resistance less likely since it would require multiple simultaneous mutations rather than single-gene changes as in conventional antibiotics [32].Given their structural diversity and multifunctionality, it is essential to understand in detail how AMPs exert their biological activity. Such knowledge provides the basis for rational design and for tailoring these molecules toward therapeutic applications.

### 3.1 Mechanism of action

AMPs act through multiple, complementary mechanisms. Beyond disrupting microbial membranes, many AMPs reach intracellular targets and/or modulate host immunity. For clarity, their activity can be grouped into membrane-targeting and non-membrane-targeting mechanisms, with additional roles in immune modulation [33]. In Gram-negative bacteria, outer-membrane lipopolysaccharides (LPS) and their stabilization by Mg^2+^/Ca^2+^ ions play a critical role in AMP binding and translocation through the so-called self-promoted uptake pathway [34].

#### 3.1.1 Membrane-targeting

**Fig. 2:**
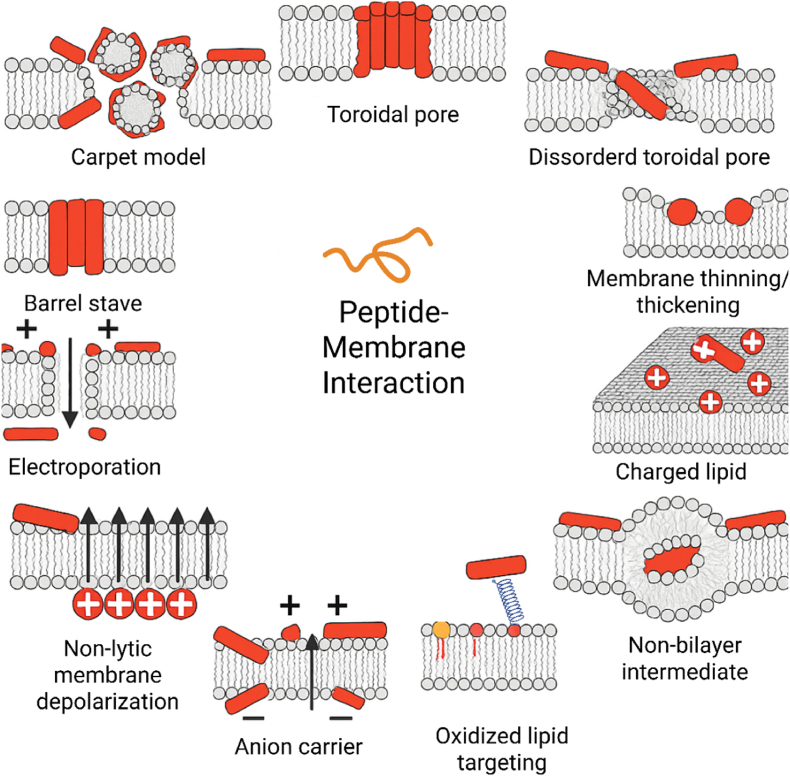
Mechanisms of peptide–membrane interaction and models of bacterial membrane disruption induced by antimicrobial peptides. The main proposed mechanisms by which AMPs compromise the integrity of the bacterial membrane are depicted. Among the classical mechanisms are the toroidal pore model, the carpet model, and the barrel stave model. Additional mechanisms are also illustrated, including the disordered toroidal pore, electroporation, membrane thinning/thickening, non-lytic membrane depolarization, anion carrier activity, charged lipid clustering, non-bilayer intermediate formation, and targeting oxidized lipids. Adapted from Nguyen et al. [40] with permission from Elsevier, copyright 2011.

The antibacterial action of AMPs typically relies on electrostatic interactions between cationic peptides and negatively charged bacterial surfaces [35]. Some bacteriocins (e.g., nisin, mesentericin) use receptor-mediated pathways, while vertebrate AMPs often interact non-specifically with membrane lipids [36]. In both Gram-positive and Gram-negative bacteria, the cytoplasmic membrane contains abundant anionic phospholipids such as phosphatidylglycerol, cardiolipin, and phosphatidylserine, while teichoic acids (Gram-positive) and LPS (Gram-negative) provide additional negative charges [37]. The classical models of membrane permeabilization are the barrel-stave, toroidal pore, and carpet models, which describe the major pathways by which AMPs compromise bacterial membranes (Fig. 2) [38,39].

**Barrel-stave model:** AMPs (e.g., alamethicin, pardaxin, protegrins) initially align parallel to the membrane, then insert perpendicularly, with hydrophobic residues interacting with lipids and hydrophilic residues forming a pore lumen [41].

**Toroidal pore model:** Peptides insert perpendicularly, induce membrane curvature, and generate transient pores that incorporate both peptides and lipid head groups, allowing selective ion passage [42].

**Carpet model:** AMPs accumulate on the surface until reaching a critical threshold, after which the membrane disintegrates into micelle-like structures without defined pores. Examples include cecropin, indolicidin, aurein, and LL-37 electroporation, membrane thinning/thickening, non-lytic depolarization, lipid clustering, and non-bilayer intermediates, likely coexist depending on lipid composition and environmental conditions [57,58].

#### 3.1.2 Non-membrane-targeting mechanisms

Some AMPs interfere with cell wall biosynthesis by binding peptidoglycan precursors, while others act on intracellular targets, including nucleic acids and essential enzymes [43]. In these cases, AMPs may accumulate inside the cell after initial membrane association, exerting lethal effects without causing extensive membrane damage at inhibitory concentrations [44].

#### 3.1.3 Immune modulation

Several AMPs regulate host immune responses. They promote leukocyte recruitment, differentiation, angiogenesis, and modulation of inflammatory pathways by controlling pro- and anti-inflammatory mediators, as well as reactive oxygen and nitrogen species [45]. Most effects involve innate immune cells such as neutrophils and macrophages, though evidence also supports interactions with adaptive immune cells, including T and B lymphocytes. The precise pathways of immune regulation remain incompletely defined [46].

### 3.2 Advantages over traditional antibiotics

AMPs have emerged as a promising alternative for treating bacterial infections, particularly multidrug-resistant (MDR) strains. This interest arises from the declining discovery of new antibiotics and the rapid development of bacterial resistance to existing ones. Unlike conventional antibiotics, AMPs are protein-based molecules that degrade into amino acids rather than potentially harmful metabolites, reducing toxicity concerns [47]. Several advantages have been identified during their development and evaluation, including synergy with antibiotics [48], a low propensity for resistance [49], sustained activity against MDR strains [50], antibiofilm effects in parallel with antimicrobial activity, a rapid bactericidal mechanism mediated by membrane disruption [51], and immunomodulatory properties already discussed in the previous section [52].

AMPs are short amino acid chains, which makes their synthesis more straightforward than that of complex antibiotic molecules. Their discovery and optimization frequently rely on *in silico* methods that predict antimicrobial activity from physicochemical properties, peptide sequences, and structural features [53]. Approximately one-third of a bacterial cell's proteins are membrane-associated, carrying out essential functions such as nutrient transport, respiration, ATP generation, and intracellular signaling. AMPs disrupt these processes by interacting with membrane proteins, sometimes without complete cell lysis. Their rapid bactericidal effect therefore results not only from membrane destabilization but also from interference with vital cellular functions features between AMPs and conventional antibiotics is presented in Table 1, highlighting their distinct mechanisms of action, broader antimicrobial potential, reduced but possible resistance development, activity against MDR pathogens, immunomodulatory capacity, toxicity considerations, and therapeutic versatility.

**Table 1:** Comparative characteristics of antimicrobial peptides and traditional antibiotics.

Characteristics	AMPs	Traditional Antibiotics	Ref.
Mechanism of action	Multiple mechanisms, mainly membrane disruption and sometimes intracellular targets	Specific mechanisms such as inhibition of protein, DNA, or cell wall synthesis	[54]
Spectrum of Activity	Broad antibacterial range; some with antifungal, antiviral, or antiparasitic effects depending on the peptide.	Generally limited to specific bacterial groups.	[55]
Development of resistance	Lower propensity compared to conventional drugs, though adaptive resistance mechanisms have been reported.	Increasing development of diverse resistance mechanisms.	[56]
Activity against multidrug-resistant strains	High potential to act against MDR pathogens due to membrane targeting.	Often limited efficacy due to existing bacterial defenses.	[31]
Toxicity and Biocompatibility	Hemolysis and cytotoxicity remain challenges; strategies under development to improve stability and safety.	Prolonged or high dose exposure associated with significant toxicity.	[57,58]
Immunomodulatory activity	Many AMPs display immunomodulatory effects that enhance host defense.	Some antibiotics also show immunomodulatory properties, though not their primary role	[31]
Potential as alternative therapeutic agents	High versatility in formulations and ongoing preclinical/clinical evaluation.	Established clinical use but increasing threat of resistance limits long term efficacy.	[59,60]

In recent years, research on antimicrobial peptides has expanded considerably, with numerous studies reporting novel sequences from diverse natural and synthetic sources, as can be seen in Table 2.

**Table 2:** AMPs reported in the last two years with demonstrated *in vivo* efficacy against Gram-negative bacteria, including their MIC values, cytotoxicity, hemolysis, and therapeutic outcomes.

AMP	MIC (μM/μg mL^−1^)	IC_50_/Cytotoxicity	Hemolysis	Gram-negative bacteria effective	*In vivo*	Ref.
LC-AMP-I1	2.5–10 μM	19.58 μM	10.13 % at 320 μM	*E. coli*, *K. pneumoniae*, *A. baumannii*, *P. aeruginosa*, *Enterobacter* spp.	1.35 log_10_ CFU/g reduction in murine thigh infection.	[69]
TC-LAR-18	2.34–9.38 μg mL^−1^	>100 μg mL^−1^	0.73 % at 100 μg mL^−1^	*A. baumannii*, *P. aeruginosa*, *E. coli.*	Up to 97.8 % bacterial reduction in murine skin infection.	[70]
TC-14	1.17–18.75 μg mL^−1^	>100 μg mL^−1^	None up to 100 μg mL^−1^	*A. baumannii*, *P. aeruginosa*, *E. coli.*	57.9 % reduction at 0.5 mg mL^−1^ and 93.1 % at 2 mg mL^−1^ in murine skin infection.	[71]
NCBP1	16–128 μM	Not toxic up to 256 μM.	<5 % up to 256 μM	*E. coli, K. pneumoniae.*	Effective in murine infection model; reduced MDR bacterial load.	[72]
S24	2–8 mg L^−1^ (≈2–8 μg mL^−1^)	>128 mg L^−1^	Minimal up to 128 mg L^−1^	*P. aeruginosa*, *A. baumannii.*	Promoted wound healing in murine skin infection with *P. aeruginosa*, *A. baumannii* and mixed strains.	[73]
Lariocidin/Lariocidin B	0.2–5 μM	IC_50_ bacterial translation: 22 nM (*E. coli* cell-free); ∼60 × higher in mammalian system.	No toxicity to human cells reported.	Broad activity including MDR *A. baumannii*, *E. coli.*	Potent efficacy in murine *A. baumannii* infection model.	[74]
T-series (e.g., T1-2, T1-5, T2-9)	3.9–62.5 μg mL^−1^	N.R.	N.R.	*E. coli*, *K. pneumoniae*, *P. aeruginosa.*	Effective in murine skin wound infection (*P. aeruginosa*).	[75]
PD45HF5	4–32 μg mL^−1^	Negligible cytotoxicity (NIH 3T3).	Minimal (HC_50_ > 4096 μg mL^−1^).	*K. pneumoniae, A. baumannii, P. aeruginosa, E. coli (MDR, ESBL).*	Potent efficacy in murine skin wound and peritonitis infection models.	[39]
AMP-24	≤12.5 μM	N.R.	N.R.	*A. baumannii*, *E. coli.*	Potent efficacy in murine skin and lung *A. baumannii* infection models.	[76]
AMP_2	≤10 μM (*E. coli*, *P. aeruginosa*, *A. baumannii*)	N.R.	Low hemolysis, minimal cytotoxicity.	*E. coli*, *P. aeruginosa*, *A. baumannii.*	Effective in murine acute peritonitis model (*E. coli* resistant strain).	[77]

∗Not reported (N.R.); Antimicrobial peptide (AMP); Lycosa coelestis antimicrobial peptide I1 (LC-AMP-I1); Tupaia chinensis LAR-18 (TC-LAR-18); Tree shrew cathelicidin peptide 14 (TC-14); Noncanonical basic peptide 1 (NCBP1); Synthetic peptide 24 (S24); Phage-display and computationally optimized antimicrobial peptide 5 (PAM-5); Lasso peptides Lariocidin and Lariocidin B (Lariocidin/Lariocidin B); Artificial intelligence–designed T-series peptides (T1-2, T1-5, T2-9, etc.); Plant-derived cyclotide Cliotide U1 (Cliotide U1); Minimum inhibitory concentration (MIC); Half maximal inhibitory concentration (IC_50_); Colony-forming unit (CFU); Multidrug resistant (MDR); Antimicrobial peptide 24 (AMP-24); Antimicrobial peptide 2 (AMP-2).

Primo et al. [61] encapsulated AMPs into chitosan nanoparticles and co-loaded them with rifampicin, which restored rifampicin's efficacy against rifampicin-resistant *Mycobacterium tuberculosis*, an effect absent in AMP-free controls. Likewise, Lu et al. [62] reported that avian cathelicidins CATH-1 and CATH-3, together with porcine PMAP-36, acted synergistically with erythromycin against *Staphylococcus aureus*, *Salmonella enteritidis*, and *Escherichia coli*. This combination reduced the minimum bactericidal concentration of erythromycin by up to 16-fold for *E. coli*, achieved complete bacterial clearance within 3 h in time-kill assays, lowered cytotoxicity compared with erythromycin alone, and delayed the emergence of resistance, highlighting the potential of AMPs as effective adjuvants.

Biofilm-associated infections represent another challenge where AMPs are particularly advantageous. Bacteria naturally adhere to surfaces and form biofilms—highly structured communities encased in an extracellular matrix that enhances tolerance to antimicrobials and immune defenses [63]. These communities often contain metabolically inactive and persistent cells that display high antibiotic tolerance [64]. AMPs are effective against biofilms because of their broad-spectrum bactericidal activity, ability to penetrate the biofilm matrix, and capacity to destabilize its structure. Masihzadeh et al. [65] showed that the synthetic peptide WLBU2 exhibited strong antibiofilm activity against *Pseudomonas aeruginosa*, significantly reducing bacterial adhesion and biofilm formation at sub-inhibitory concentrations (1/8–1/4 × MIC) and dispersing up to 92 % of preformed carbapenem-resistant biofilms at 4 × MIC.

Moreover, AMPs are readily degraded by intestinal proteases during *in vivo* applications, limiting tissue damage to the host. Representative examples include defensins, cathelicidins, and members of the regenerating islet-derived (Reg) III family (α, β, γ). In addition, several peptides influence immune function and modulate cellular signaling, thereby supporting both innate and adaptive responses [66]. Silva et al. [67] showed that clavanin-MO exhibits both antimicrobial and immunomodulatory functions. In macrophages, it increased the production of the anti-inflammatory cytokine interleukin-10 while suppressing the pro-inflammatory cytokines interleukin-12 and tumor necrosis factor alpha. In murine models, the peptide promoted rapid leukocyte recruitment and stimulated the release of granulocyte-macrophage colony-stimulating factor, interferon gamma, and monocyte chemoattractant protein-1. These combined effects accelerated infection resolution and protected mice from lethal infections caused by multidrug-resistant pathogens. Torres et al. [68] demonstrated that encrypted peptides derived from non-immune proteins exert. both antimicrobial and immunomodulatory functions. Nearly 90 % of the tested peptides modulated immune mediators, either enhancing pro-inflammatory cytokines such as tumor necrosis factor alpha and interleukin-6 or reducing them under specific conditions, while also increasing the chemokine monocyte chemoattractant protein-1. In preclinical models, selected peptides reduced bacterial infections by up to four orders of magnitude, supporting their role as multifunctional host defense agents.

Although AMPs display multiple attributes that support their use as next-generation antimicrobials, they are not exempt from drawbacks. These limitations—ranging from production hurdles to stability issues—must be critically addressed before their therapeutic promise can be fully realized. Section 3.3 outlines the main constraints that still hinder their clinical translation.

### 3.3 Limitations of antimicrobial peptides

Although AMPs offer advantages over conventional antibiotics, they still face limitations that restrict their clinical application. The main challenges include low production yields, lengthy development times, and high manufacturing costs [78]. Large-scale direct extraction of natural AMPs is unfeasible because they are small molecules and chemical synthesis have been employed; however, peptide production remains expensive, and direct expression of AMP genes in microorganisms presents difficulties due to potential toxic effects in host cells (typically bacteria and fungi) [79]. *In silico* approaches have contributed to reducing costs and accelerating the discovery of new AMPs [86]. Gene expression in the form of proteins has also been explored, but despite showing significant *in vitro* activity, their *in vivo* efficacy is often limited by enzymatic degradation and physiological pH variations, which reduce circulation time and antimicrobial activity [80].

Regarding safety, AMPs are generally less harmful to human cells than many antibiotics, which represents an advantage. However, some peptides present limitations associated with nonspecific interactions with eukaryotic membranes, which may cause hemolysis, as well as undesired immunomodulatory effects [81,82]. Therefore, although AMPs present an overall more favorable safety profile, they require structural optimization [83].

Several strategies have been proposed to overcome these limitations, including heterologous expression systems, synthetic modifications, and encapsulation technologies to improve peptide stability [84]. Chemical modifications such as cyclization, incorporation of D-amino acids, and acetylation have been shown to increase bioavailability and resistance to degradation [85].

Despite these challenges, some AMPs have already been approved by the United States Food and Drug Administration (FDA) for clinical use, demonstrating efficacy against bacterial and viral infections [84]. Among them, gramicidin, derived from *Bacillus brevis*, and polymyxins, produced by *Paenibacillus polymyxa*, exhibit strong antibacterial activity: the former is used mainly for localized infections, whereas the latter have both local and systemic applications [86]. Vancomycin is a widely used systemic antibiotic effective against Gram-positive bacteria, particularly in drug-resistant infections [87]. Daptomycin is produced by *Streptomyces roseosporus* and is administered intravenously for complicated skin and skin structure infections, *Staphylococcus aureus* bacteremia, and right-sided infective endocarditis [88].

Clinically, although more than 80 % of natural AMPs display antibacterial activity, only a minority achieve efficacy against Gram-negative bacteria [89,90]. Among FDA-approved AMPs, colistin remains the only one with clear selectivity against these pathogens, and its current use is restricted to last-resort therapy due to nephrotoxicity and neurotoxicity [91]. Other peptides, such as pexiganan, a magainin analog evaluated as a topical cream for diabetic foot infections, reached Phase III but was not approved because it offered no advantages over existing therapies [92]. LL-37, a human cathelicidin currently in Phase II, has been investigated for diabetic foot ulcers owing to its antimicrobial and immunomodulatory properties; however, its clinical development is hampered by toxicity, susceptibility to proteases, and high production costs, which has prompted the search for truncated or more stable derivatives [93].

Despite promising *in vitro* activity and a relatively small number of preclinical studies, translation into effective clinical therapies against Gram-negative infections remains uncommon. Nevertheless, advances in nanotechnology are offering strategies to overcome many of the obstacles associated with AMP delivery and stability. The following section examines how nanoplatforms have been engineered to enhance AMP bioavailability, prolong circulation, and enable targeted therapeutic applications.

## 4 Nanoplatforms for peptide-mediated delivery and targeting

### 4.1 Lipid-based nanoparticles

Lipid-based nanoparticles are drug delivery systems primarily composed of lipids and surfactants, capable of encapsulating, incorporating, or surface-loading a wide variety of therapeutic agents — ranging from small molecules and peptides to nucleic acids — protecting them from enzymatic degradation and enhancing their transport across cellular membranes [94,95]. The morphology and stability of these nanostructures depend on multiple factors, including the type of lipid employed, the nature of the active compound and surfactant, as well as the production method, loading capacity (LC), encapsulation efficiency (EE), and final particle size [96]. These variables also affect the micropolarity and internal microviscosity of the lipid matrix, thereby modulating the drug release kinetics [97]. Several nanoplatforms have been developed, including liposomes, solid lipid nanoparticles (SLNs), nanostructured lipid carriers (NLCs), cubosomes and niosomes [98,99]. Although all share a vesicular-type lipid organization, each differs in the specific composition of its lipid components, directly influencing drug LC, EE, and release profiles [100].

An often-overlooked aspect of lipid-based systems is their phase behavior, which arises from the interplay between amphiphile composition and processing conditions [101]. Depending on the amphiphile composition and processing conditions, different lyotropic phases can be generated: lamellar bilayers (Lα) forming liposomes, inverse hexagonal structures (HII) yielding hexosomes, or inverse bicontinuous cubic arrangements (QII) forming cubosomes. [102]. These lipid arrangements are dynamic rather than fixed. Their structural outcome depends on multiple variables: lipid composition, hydration level, temperature, and pressure can shift bilayer systems toward non-lamellar phases [103]; local protonation states and pH gradients alter headgroup charge, membrane curvature, and vesicle morphology, inducing migration, deformation, or phase transitions [104]; and molecular features such as chain length, degree of unsaturation, and cholesterol content, together with processing parameters including pH, ionic strength, mixing conditions, and microfluidic formulation, direct self-assembly into micelles, liposomes, cubosomes, or hexosomes [105]. A useful geometric descriptor for these transitions is the critical packing parameter (CPP), defined as $\text{CPP}=\frac{V}{\alpha *l}$, where V is the hydrophobic chain volume, α the effective headgroup area, and l the hydrophobic chain length in the molten state. According to this model, CPP <1/3 favors spherical micelles, 1/3–1/2 cylindrical micelles, ∼1 lamellar bilayers, and p > 1 inverse phases [106].

#### 4.1.1 Liposomes

Liposomes are phospholipid bilayer vesicles that closely mimic biological membranes and can host cargoes in aqueous and lipid domains, but their defining features are governed by bilayer composition and structure [107,108]. This architecture enables the encapsulation of both amphiphilic, lipid-soluble compounds and hydrophilic substances, allowing for controlled release to improve the efficacy of nutraceuticals, pharmaceuticals, and other bioactive compounds [[109], [110], [111]]. Liposomes are primarily composed of phospholipids such as phosphatidylcholine (PC), phosphatidylethanolamine (PE), phosphatidylglycerol (PG), phosphatidylinositol, and sphingomyelin, with soy lecithin being the most widely used source [112]. To enhance their stability, they are often combined with modulators including cholesterol or phytosterols, as well as non-ionic surfactants (Tween, Span) and polymers such as chitosan, which regulate bilayer fluidity, elasticity, and permeability. The final structure and functionality of liposomes depend on the interplay of these components together with parameters such as the hydrophilic–lipophilic balance and CPP [113]. According to Steffes et al. [114], lipids with an inverted-cone geometry, such as dioleoylphosphatidylethanolamine (DOPE) and glyceryl monooleate (GMO), introduce a negative spontaneous curvature (C_0_ < 0) that destabilizes Lα and favors nonlamellar organizations, including the HII and inverse QII phases. The incorporation of such curvature-promoting amphiphiles alters bilayer packing and reduces the capacity of liposomes to maintain stable lamellar structures, often leading to diminished drug retention and accelerated phase transitions.

The physicochemical traits of phospholipids determine bilayer performance. The degree of acyl-chain saturation and the associated phase transition temperature define bilayer fluidity and stability [115]. Cholesterol modulates these properties by tightening phospholipid packing, lowering membrane permeability, and improving vesicle resistance to aggregation, while at the same time influencing the lamellar-to-inverted hexagonal phase transition that underlies endosomal disruption and drug release [116]. In parallel, the choice of helper lipids strongly affects structural geometry: cylindrical lipids such as distearoylphosphatidylcholine or dioleoylphosphatidylcholine promote stable bilayers, whereas cone-shaped DOPE induces fusogenic non-lamellar phases that enhance transfection efficiency and endosomal escape, though often at the expense of storage stability [117]. In liposomal formulations, surface interactions with biological fluids govern their fate *in vivo*. Upon systemic administration, circulating proteins rapidly adsorb to the lipid bilayer, forming a dynamic protein corona that redefines colloidal stability and immune recognition [118]. This adsorbed layer constitutes a new biological identity, ultimately dictating biodistribution, immunogenicity, and clearance pathways [119]. The specific proteins recruited can expose functional motifs that facilitate receptor-mediated uptake, as shown for apolipoproteins binding to peptide-decorated liposomes [120].

Among the most relevant synthesis methods is the thin-film hydration technique, widely adopted due to its simplicity and industrial scalability, although it presents limitations such as heterogeneous liposome sizes and relatively low EE [121]. Another notable approach is reverse-phase evaporation, which provides moderate EE for hydrophilic and amphiphilic compounds by forming emulsions followed by sonication, although it requires careful removal of organic solvents [122]. More recently, microfluidics-based methods represent a significant technological advancement, offering precise control over liposome size and uniformity. Despite their high cost and technical complexity, they are a promising alternative for large-scale production [113,123,124].

Recent evidence illustrates how control over lipid composition and interfacial organization translates design intent into therapeutic function. Sanches et al. [125] reported that in rhamnolipid/POPC/cholesterol formulations, the inclusion of cholesterol increased EE and prolonged release, transforming an otherwise inactive peptide into an active agent against *E. coli* and *S. aureus*, underscoring that lipid choice is not a secondary detail but a determinant of antimicrobial potency. Beyond demonstrating the efficacy of a single system, this study highlights how lipid composition can modulate encapsulation, stability, and the bioavailability of AMPs, suggesting that the lipids employed are not mere excipients but critical determinants of antimicrobial activity. The next challenge lies in translating this protective and delivery capacity into clinical settings against resistant pathogens, where AMPs require an environment that maximizes their intracellular activity. In another study, Hemmingsen et al. [126] developed cationic liposomes loaded with a synthetic AMP mimetic, which slowed release and extended antimicrobial activity against biofilms of *S. aureus*, *E. coli*, and *P. aeruginosa*, while also reducing NO production in macrophages, thereby providing a dual antimicrobial and immunomodulatory effect. This work validates the utility of liposomes for the protection and delivery of peptidomimetics and further opens a line of research into their role as dual platforms capable of combining antimicrobial and immunomodulatory functions. In clinical practice, such dual action is particularly promising for chronic wounds, where the persistence of biofilms and uncontrolled inflammation often hinder healing.

The studies reviewed indicate that liposomes represent a versatile platform to enhance the performance of antimicrobial peptides and their analogs, providing protection against degradation, sustained release, and, in some designs, additional immunomodulatory properties. Nonetheless, relevant limitations remain, including burst release in fusogenic phases, instability in protein-rich environments, and the lack of comparative evaluations that would allow for the definition of stronger design principles. Progress toward clinical translation will require standardized characterization parameters, direct comparisons using the same peptides across different formulations, and the adoption of scalable manufacturing approaches under quality-by-design frameworks, with the ultimate goal of transforming liposomes into reproducible and clinically viable platforms against resistant bacterial infections.

#### 4.1.2 Solid lipid nanoparticles

SLNs are characterized by their spherical shape and nanometric size, with lipids remaining in a solid state at both room and body temperatures [127]. Composed of fatty acids, triglycerides, steroids, and crystallized waxes combined with emulsifiers and surfactants, SLNs exhibit high surface area and favorable zeta potential (ZP) [128,129]. In these systems, drug loading, EE, and release kinetics are influenced by particle size, morphology, surface charge, stability, and polymorphism [130,131]. The use of solid lipids with different chain lengths and melting points influences particle size, dispersibility, and encapsulation efficiency, while rapid cooling or inappropriate lipid selection may induce unstable crystal forms that reduce drug retention [132]. The lipid matrix undergoes polymorphic transitions (α-β′-β), and these structural rearrangements can expel the encapsulated compound or modulate its diffusion. Oehlke et al. [133]*,* demonstrated that such polymorphic changes directly affect the release or activity of encapsulated antioxidants, where drug expulsion is often associated with transition into more stable β forms. By tailoring lipid blends and cooling conditions, these transitions can be controlled, reducing burst release and enabling a more sustained delivery profile.

SLNs can be produced via two main approaches, classified according to the amount of energy applied during synthesis. High-energy methods involve equipment such as high-pressure homogenizers, high-shear homogenizers, and ultrasonicators, enabling efficient particle size reduction [96]. In contrast, low-energy methods rely on gentler processes such as magnetic stirring, microemulsion formation, thermal phase inversion, and solvent-based techniques like solvent diffusion or injection, which are particularly suitable for heat-sensitive or poorly stable compounds [134].

**Fig. 3:**
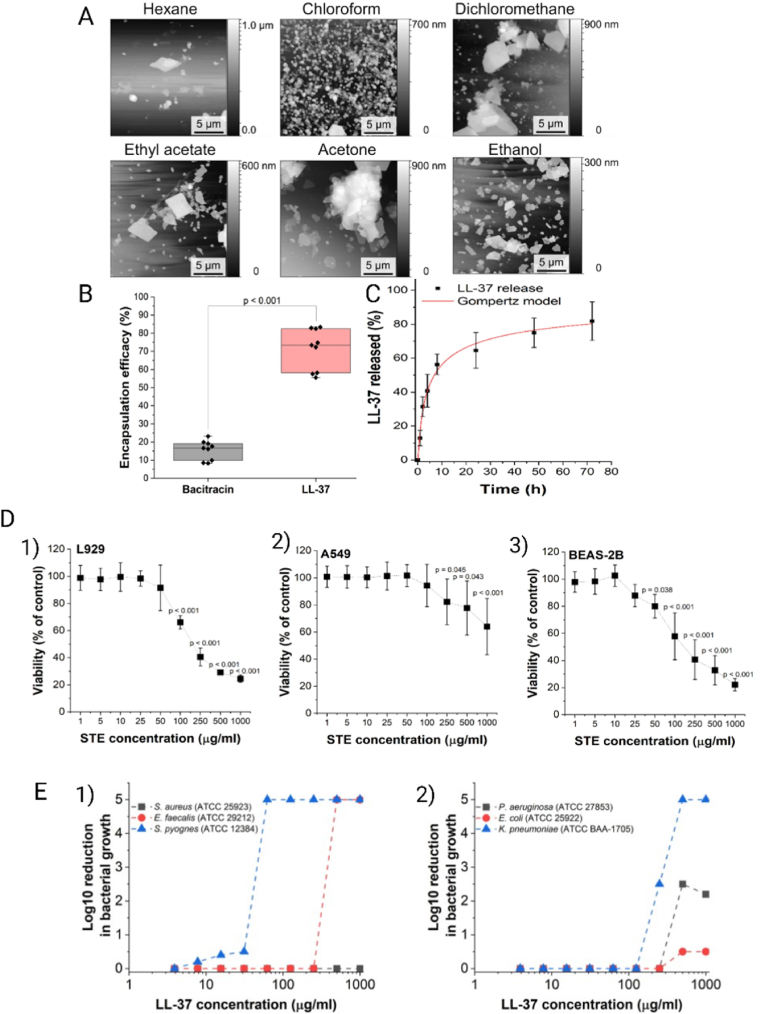
**A)** AFM images illustrate the morphology of stearic acid-based nanoparticles synthesized using a range of organic solvents with varying degrees of water miscibility. The choice of solvent impacted particle uniformity and shape, with chloroform yielding the most homogeneous and spherical structures. **B)** EE (%) of bacitracin A and LL-37 in stearic acid-based nanoparticles, highlighting a markedly higher loading capacity for LL-37 compared to bacitracin A. **C)***In vitro* cumulative release profile of LL-37 from stearic acid nanoparticles over 72 h, fitted to a Gompertz model, indicating sustained peptide release. **D)** Evaluation of the cytotoxicity of unloaded stearic acid nanoparticles using three different cell lines— **1)** murine fibroblasts (L929), and human pulmonary epithelial cells **2)** A549 and **3)** BEAS-2B after 24 h of incubation. **E)** Log reduction in bacterial growth for Gram-positive [1] and Gram-negative [2] bacteria in the presence of LL-37. Reproduced with permission [135]. Copyright 2024, Elsevier.

Reczyńska-Kolman et al. [135] developed stearic acid-based SLNs using an emulsification/solvent-diffusion method for the delivery of Bacitracin A and LL-37. Solvent choice strongly influenced morphology, with chloroform yielding the most homogeneous and spherical particles, as shown in atomic force microscopy (AFM) images (Fig. 3A). EE was markedly higher for LL-37 (71 ± 10 %) compared to Bacitracin A (15 ± 5 %), accompanied by a shift in ZP from −15.4 mV in unloaded SLNs to +10.5 mV in LL-37-loaded formulations, confirming effective association with the lipid matrix (Fig. 3B). Release studies revealed a sustained profile, with over 80 % cumulative release at 72 h following a Gompertz model (Fig. 3C). Cytocompatibility assays showed no significant toxicity of unloaded SLNs up to 50 μg mL^−1^ in L929 fibroblasts and A549 and BEAS-2B epithelial cells (Fig. 3D). Antibacterial testing against Gram-negative bacteria indicated that free LL-37 displayed only limited activity against *E. coli* and *K. pneumoniae*, while LL-37-loaded SLNs moderately improved efficacy, though complete inhibition was not achieved (Fig. 3E). These results illustrate how solvent selection, the interaction of cationic peptides with the lipid matrix, and the crystalline organization of the solid core govern encapsulation efficiency, release kinetics, and antimicrobial performance in SLNs. Although activity against Gram-negative pathogens remains modest, this approach provides a framework for further optimization through tailored lipid blends or surface modifications that favor intracellular action. Importantly, the high crystallinity of the solid lipid matrix often promotes drug expulsion during storage or polymorphic transitions; this limitation has led to the development of NLCs, in which partial substitution of solid lipids with liquid lipids introduces lattice imperfections that increase loading capacity and enable smoother release profiles, as discussed in the following section [97,136].

#### 4.1.3 Nanostructured lipid carriers

NLCs combine solid and liquid lipids to form an imperfect matrix that improves drug loading capacity — owing to the higher solubility of actives in the oily component — and reduces the water content of the suspension, thereby minimizing the premature release of active compounds during storage [128]. Although NLCs pose a greater challenge for surface functionalization, their hybrid composition offers unique flexibility to encapsulate poorly water-soluble agents and achieve more stable release profiles tailored to diverse therapeutic applications [137,138]. The synthesis methods employed for NLCs are essentially the same as for SLNs, such as high-pressure homogenization or emulsification, with adjustments in the lipid types and ratios to favor the formation of the characteristic amorphous matrix of NLCs [139]. Thanks to their composition and structural properties, lipid-based nanoparticles offer exceptional biocompatibility and remarkable mimicry of cellular membranes, facilitating fusion or internalization into target cells, thereby enhancing the intracellular delivery of therapeutic peptides [[140], [141], [142]]. Their amphiphilic nature allows for dual encapsulation strategies: hydrophilic compounds can be incorporated into the aqueous compartments or core, while hydrophobic or amphiphilic molecules integrate into the lipid bilayer or outer lipid matrix, supporting stable and controlled release of the active compound [143,144]. Additionally, these structures protect peptides from enzymatic degradation and extreme pH variations, thereby increasing their stability and bioavailability [145].

Despite their significant advantages, lipid-based nanoparticles present challenges that may limit their therapeutic performance. Aggregation and vesicle fusion in liposomes, as well as premature payload leakage, compromise -controlled release and can trigger burst drug release events [146]. SLNs often exhibit high matrix crystallinity, which may progressively expel the peptide and generate non-uniform release profiles, while NLCs partially overcome this drawback but can still undergo phase separation and particle size fluctuations [147,148]. In fact, the polymorphic transitions of solid lipids (α–β′–β) largely determine drug retention or expulsion in SLNs, whereas the hybrid blending of solid and liquid lipids in NLCs disrupts crystallinity and mitigates these effects. Overall, composition and processing history dictate the structural organization and, consequently, the therapeutic performance of lipid nanocarriers [149,150].

Building on these principles, Chetty et al. [151] developed multifunctional enzyme-responsive NLCs for the co-delivery of vancomycin (VCM) and the cationic AMP RKKKRLLRKKC. The optimized formulation, composed of triglycerol monostearate (TGMS) as the solid lipid and farnesol (FAN) as the liquid lipid, achieved favorable physicochemical parameters (149 nm, polydispersity index (PDI) 0.07, and an EE of ∼86 %) and demonstrated sensitivity to infection-associated enzymes, such as matrix metalloproteinases and bacterial lipases. Enzyme-triggered destabilization was validated both structurally and functionally, correlating with controlled drug release. Antimicrobial assays showed that VCM-AMP-TF-NLCs reduced the MIC of vancomycin up to eight-fold against *E. coli*, while *in vivo* treatment lowered bacterial burden nearly nine-fold and alleviated tissue injury compared with free VCM.

Beyond confirming the efficacy of a single formulation, this study underscores the potential of NLCs as adaptive carriers capable of combining AMP stabilization with enzyme-triggered release in infection microenvironments. In contrast to liposomes, where clinical translation remains the main challenge, and SLNs, which are limited by crystallinity and scalability, NLCs emerge as platforms with the capacity to integrate into combined therapeutic strategies and infection models that replicate specific enzymatic profiles, paving the way for more personalized treatment schemes against resistant Gram-negative pathogens.

#### 4.1.4 Cubosomes

Cubosomes are self-assembled nanoparticles composed of amphiphilic lipids, typically monoolein (MO) or phytantriol, and stabilized by polymers such as Pluronic F127 [152]. Under aqueous conditions, these components spontaneously organize into a QII phase, whose colloidal dispersions are known as cubosomes. This phase features a three-dimensional matrix of interconnected aqueous channels separated by lipid bilayers, arranged according to three main symmetries: double diamond, gyroid, and primitive [153]. Such architecture enables the simultaneous encapsulation of hydrophobic, hydrophilic, and amphiphilic compounds [152,154]. while the channel geometry can be tuned through lipid composition, hydration, and temperature, thereby modulating encapsulation and release properties [155]. Recent studies also revealed that molecular chirality can be transferred to these nanostructures, expanding their potential in advanced delivery applications [156].

The preparation of cubosomes commonly involves emulsifying the molten lipid in excess water in the presence of a stabilizer, followed by high-energy processes such as sonication or high-pressure homogenization [157]. These procedures fragment the bulk cubic phase into discrete nanoparticles while preserving the internal organization. Both top-down approaches (dispersing pre-formed cubic phases) and bottom-up methods (direct precipitation from lipid solution) have been described [158].

Among their main advantages, cubosomes demonstrate high colloidal stability, biocompatibility, and versatility, providing protection against enzymatic degradation and enabling the controlled and sustained release of encapsulated agents. Their internal organization enables the incorporation of both hydrophobic drugs (within the lipid domains) and hydrophilic drugs (in the aqueous channels), thus facilitating the delivery of small molecules, peptides, proteins, or even nucleic acids [155,159,160]. In addition, their surfaces can be functionalized with targeting ligands to achieve site-specific delivery, thereby enhancing therapeutic efficacy and minimizing off-target effects [161].

However, cubosomes still present notable challenges for clinical application, including restricted loading of large biomolecules due to limited pore size, and sensitivity to physiological conditions such as pH and ionic strength, which may compromise stability and drug release. Large-scale production remains difficult owing to the need for high-energy processes, while long-term stability can be affected by lipid oxidation or ester hydrolysis, particularly in monoolein-based systems [154].

**Fig. 4:**
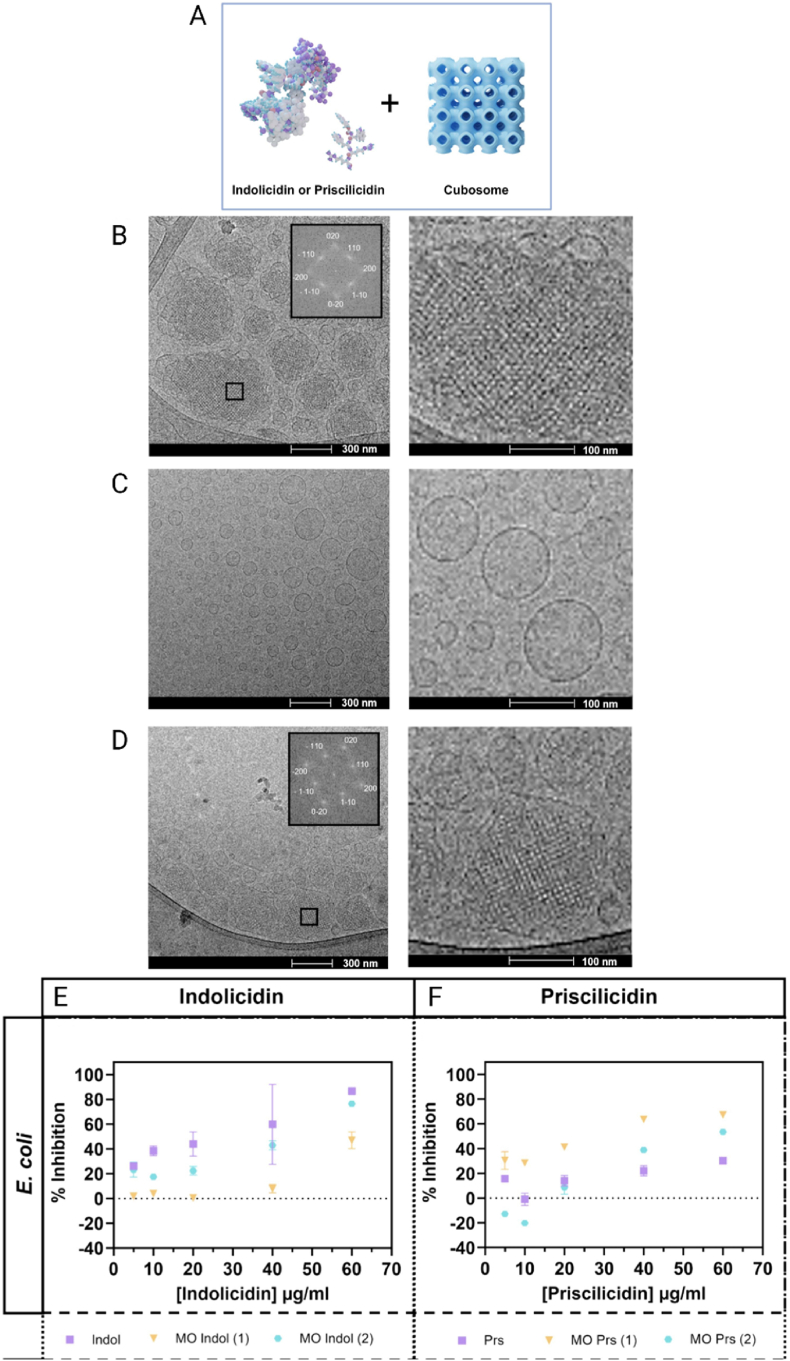
**A)** Schematic representation of Indolicidin (Indol), Priscilicidin and cubosomes. Cryo-TEM Images of **B**) MO reference sample, **C**) MO-Indolicidin (1 mol%) loaded cubosomes, and **D**) MO-Priscilicidin (1 mol%) loaded cubosomes. All samples shown are in Milli-Q water. Inserts: Fast Fourier transform (FFT) analysis for ordered particles MO and MO Prs (1 mol%). Measured optical density (OD) values calculated as % inhibition relative to the concentration of AMP against two bacterial strains for unloaded, 1 mol% [1] and 2 mol% [2] loaded peptides. **E**) Indolicidin in salt samples against *E. coli* and **F**) Priscilicidin in salt samples against *E. coli* (Salt; 150 mM NaCl). In cases where there are no error bars, the error bars are smaller than the symbol. Reproduced with permission [162]. Copyright 2025, Elsevier.

Aiming to overcome enzymatic degradation process of AMPs, Lakic et al. [162] designed cubosomes based on MO to encapsulate Indolicidin (Indol), a natural cationic AMP, and Priscilicidin (Prs), a shorter synthetic analog developed to preserve antimicrobial activity while reducing production costs. The authors reported that the addition of 150 mM NaCl prevented structural collapse during peptide loading, which markedly increased EE to 96 and 99 % % for Indol and Prs, respectively, compared with 32 and 73 % in cubosomes prepared in Milli-Q water (Fig. 4A). Cryogenic transmission electron microscopy (Cryo-TEM) analysis revealed that Indol disrupted the cubic arrangement even at 1 mol% loading, producing mostly unstructured vesicles in contrast to the ordered particles observed in the control sample (Fig. 4B–C). In contrast, Prs preserved the cubic organization under the same conditions (Fig. 4D). Dynamic light scattering measurements indicated particle sizes ranging from 145 to 521 nm, and zeta potential values became strongly positive, up to +36 mV, consistent with the incorporation of cationic AMPs. Antibacterial assays against *E. coli* showed that Indol encapsulated in NaCl-containing cubosomes was less effective than the free peptide and that increasing the loading to 2 mol% further reduced efficacy (Fig. 4E). In contrast, Prs encapsulated at 1 mol% in the presence of NaCl exhibited superior antibacterial activity compared with the free peptide at all concentrations tested, although efficacy decreased when the loading was increased to 2 mol% (Fig. 4F). The authors attributed this decline to restricted peptide mobility within the lipidic matrix, which limits interactions with bacterial membranes.

This study demonstrates that lipid composition, ionic strength of the medium, and peptide identity are decisive factors governing the structural stability and antimicrobial performance of cubosomes. Beyond the specific findings with Indol and Prs, the work highlights that the successful design of AMP-loaded cubosomes requires preserving the cubic phase, balancing surface charge to maintain peptide mobility, and tailoring formulations to physiologically relevant conditions. These principles provide a framework for extending the application of cubosomes as carriers for peptides against resistant Gram-negative bacteria, provided that challenges related to scale-up and long-term stability are resolved.

#### 4.1.5 Niosomes

Niosomes are self-assembled nanovesicles primarily composed of non-ionic surfactants and frequently include cholesterol or other lipids. Structurally reminiscent of liposomes, niosomes possess the unique ability to encapsulate both hydrophilic molecules within their aqueous core and lipophilic compounds within their bilayer membrane [163]. Their structural and functional performance depends strongly on the physicochemical characteristics of the surfactants, including hydrophile–lipophile balance, alkyl-chain length, and headgroup type, which govern vesicle size, curvature, encapsulation efficiency, and overall stability [164,165]. In addition, surface modification strategies such as PEGylation have been shown to enhance colloidal stability and prolong circulation time by reducing uptake by the reticuloendothelial system [166].

Among the most notable advantages of niosomes is their enhanced chemical and physical stability compared to liposomes, combined with favorable features such as biodegradability, biocompatibility, and ease of laboratory preparation [167,168]. Niosomes also exhibit low toxicity, with the potential for prolonged circulation time and controlled, targeted release of their payloads [169]. This, in turn, can significantly improve bioavailability while minimizing adverse effects. Their flexible composition further allows for the inclusion of various functional agents, enabling modulation of vesicle size and stability to meet specific therapeutic needs [170].

Despite these advantages, the cost-effectiveness and scalability of niosomes are strongly method-dependent. For instance, thin-film hydration may limit industrial production due to solvent residues and stability concerns, whereas microfluidization or extrusion can generate more reproducible batches, albeit at higher expense [171]. Although niosomes show several advantages, the vesicular aggregation or fusion during storage may compromise formulation stability [172].

A range of preparation strategies have been developed for niosomes, with the thin-film hydration method being among the most widely used. This approach involves dissolving lipid components in an organic solvent, evaporating the solvent to form a thin film, and subsequently hydrating the film with an aqueous phase [173]. Other techniques include ether injection, which consists of injecting a surfactant solution into a heated aqueous/ether media; sonication, used to reduce vesicle size; bubble method; microfluidization; reverse-phase evaporation; and multiple membrane extrusion [174]. Each of these methodologies offers opportunities to fine-tune vesicle size, lamellarity, and EE according to the requirements of the intended formulation.

**Fig. 5:**
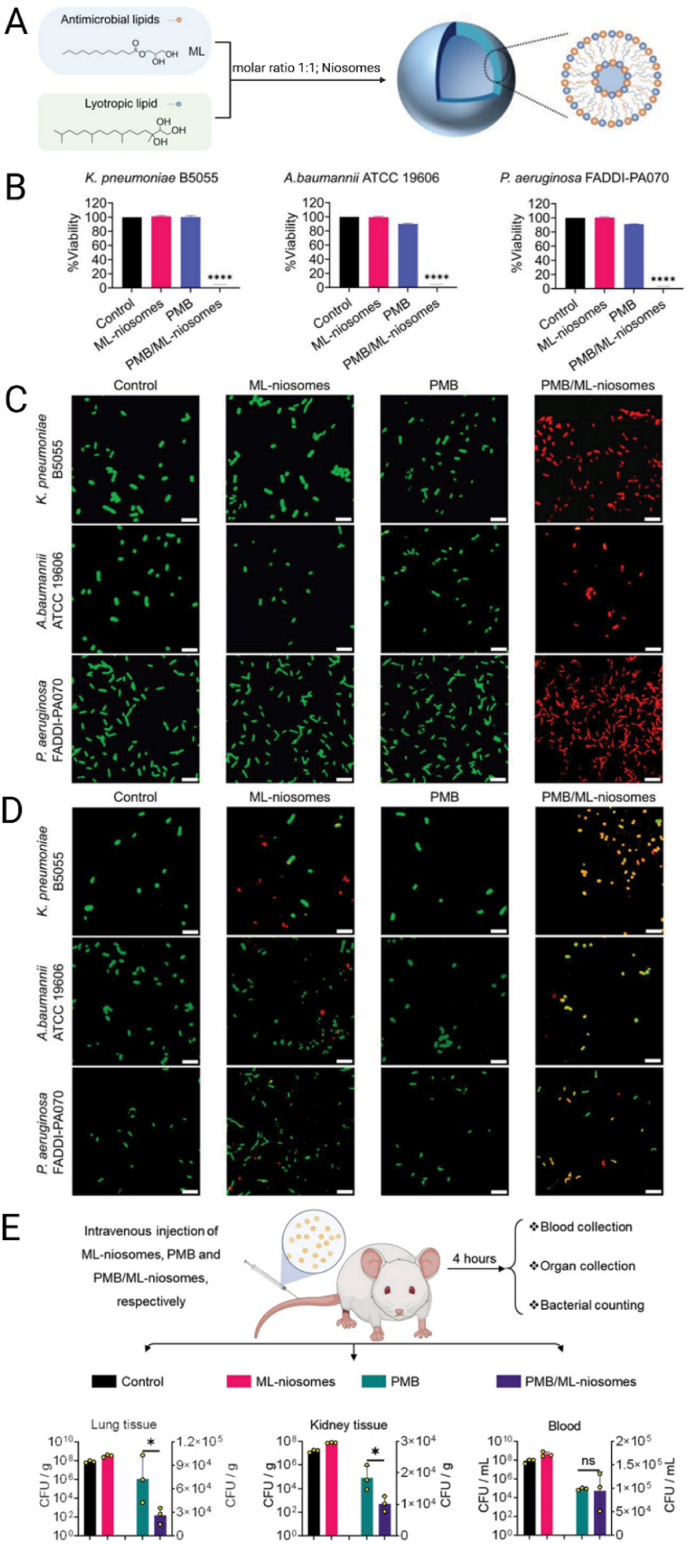
**A)** Chemical structures of monolaurin and phytantriol and their co-assembly (m = 1) forming ML-niosomes. **B)** Viability of *K. pneumoniae*, *A. baumannii*, and *P. aeruginosa* after 24 h treatment with ML-niosomes (64 μg mL^−1^), polymyxin B (PMB, 1 × MIC), and PMB/ML-niosomes. ∗∗∗∗p < 0.0001 vs. PMB. **C)** Live/dead staining: green (intact membranes, SYTO 9), red (permeabilized membranes, propidium iodide (PI)). **D)** CLSM images of bacteria (labeled green with Cell Brite Fix 488) and ML-niosomes and PMB/ML-niosomes (labeled red with octadecyl rhodamine B chloride); yellow indicates interaction (n = 3). Scale bar: 5 μm. **E)***In vivo* efficacy in a murine infection model: CFU counts in lung, kidney, and blood 4 h after treatment with ML-niosomes, free PMB, or PMB/ML-niosomes. ∗p < 0.05 compared to PMB-treat group; ns, not significant. Reproduced with permission [175]. Copyright 2023, Wiley.

Expanding on these principles, Lai et al. [175] introduced monolaurin-based niosomes (ML-niosomes) that integrate antimicrobial and lyotropic lipids to destabilize bacterial membranes and capsular polysaccharides. Incorporation of polymyxin B (PMB) generated PMB/ML-niosomes with mean diameters of 131 nm, PDI 0.18, and a zeta potential of −21 mV, while Cryo-TEM confirmed their spherical morphology and well-ordered internal structure (Fig. 5A). Antimicrobial assays demonstrated clear synergistic activity, with reductions of up to nine log units in *K. pneumoniae* B5055, *A. baumannii* ATCC 19606, and *P. aeruginosa* FADDI-PA070 after 24 h (Fig. 5B). Confocal laser scanning microscopy (CLSM) showed that PMB/ML-niosomes selectively permeabilized bacterial membranes, as evidenced by red fluorescence in live/dead staining (Fig. 5C), and dual labeling confirmed preferential interaction with bacterial surfaces (Fig. 5D). *In vivo* evaluation in a murine bloodstream infection model further validated efficacy, with significant reductions in bacterial loads in lungs and kidneys after treatment compared with free PMB (Fig. 5E). These findings underscore that the therapeutic performance of niosomes is not solely defined by drug loading but by the interplay between surfactant composition, vesicle architecture, and their interactions with bacterial envelopes. For antimicrobial peptides and antibiotics alike, the rational design of niosomes should therefore emphasize surfactant selection and stabilization strategies that prevent vesicle aggregation during storage and ensure reproducibility across diverse Gram-negative pathogens.

Beyond lipid-based systems, polymeric nanoparticles provide controllable degradation and functionalization—whether passive through the enhanced permeability and retention (EPR) effect or active via ligand attachment—thus expanding the design space for peptide stability, release kinetics, and targeted delivery [176].

### 4.2 Polymeric nanoparticles

Polymeric nanoparticles represent highly versatile drug delivery systems that can be designed from natural or synthetic polymers, either as solid nanospheres or as nanocapsules with liquid cores [177]. These systems enable the encapsulation of therapeutic peptides, protecting them from enzymatic and chemical degradation while improving their solubility, stability, and *in vivo* half-life. Moreover, they allow for increased drug concentration at the target site, thereby enhancing therapeutic efficacy while minimizing systemic side effects [178,179].

In terms of composition, natural polymers such as cellulose, chitosan or polypeptides stand out for their biocompatibility and biodegradability, making them widely used in biomedical applications and tissue engineering [[180], [181], [182]]. However, they present limitations such as batch-to-batch variability, potential immunogenicity, and limited chemical modification capacity [183]. In contrast, synthetic polymers such as polylactic acid (PLA), poly(lactic-co-glycolic acid) (PLGA), and block copolymers offer greater versatility [184]. Their chemistry can be finely tuned in terms of molecular weight, degradation rate, hydrophobicity, and functionalization of terminal groups, enabling precise control over release kinetics, peptide stability, and interactions with specific tissues [[185], [186], [187]].

Polymeric nanoparticles also enable passive targeting by exploiting the EPR effect, as well as active targeting through the conjugation of antibodies, peptides, or ligands that promote receptor-mediated endocytosis [188]. This specific targeting capability ensures that the therapeutic peptide reaches the target cells directly, enhancing its efficacy while minimizing damage to healthy tissues. Additionally, their release profile can be tuned to maintain sustained therapeutic concentrations and to avoid burst release, which could compromise treatment safety [[189], [190], [191]].

Nevertheless, despite their advantages, these systems also face significant challenges. One of the main drawbacks is the potential toxicity associated with certain synthetic polymers and their degradation byproducts, especially if they are not fully biocompatible or tend to accumulate in tissues [192,193]. Furthermore, some manufacturing processes require the use of organic solvents, which poses risks to both process safety and the stability of the encapsulated active compound. Another important limitation is the low stability of certain systems in acidic biological environments, which can compromise therapeutic efficacy, as observed in chitosan nanoparticles prepared by ionic gelation [194].

Among the most commonly employed techniques are solvent evaporation, which produces well-defined nanoparticles but requires rigorous solvent removal [193]; nanoprecipitation, which enables the rapid and simple generation of small, homogeneous particles [195]; ionic gelation, particularly useful for polymers like chitosan, which forms nanoparticles through crosslinking with multivalent anions such as tripolyphosphate [61]; and emulsion or mini-emulsion polymerization methods, which allow for high loading of hydrophobic or hydrophilic peptides and precise control over particle size and distribution [196].

**Fig. 6:**
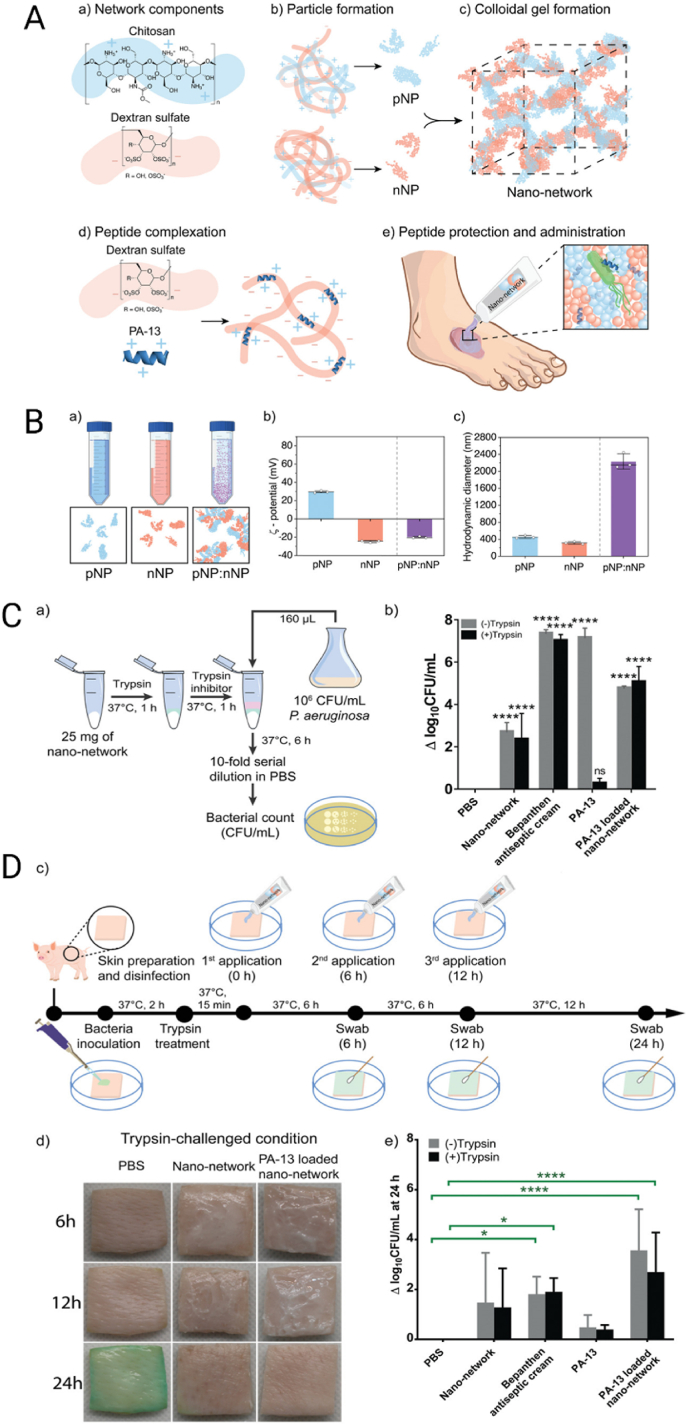
**A)** Schematic of nano-network formation from chitosan (CS) and dextran sulfate (DS): **a)** CS (5:3) formed positively charged NPs (pNPs), and DS (3:5) formed negatively charged NPs (nNPs). **b–c)** Mixing pNPs and nNPs yielded colloidal gel aggregates. **d)** PA-13 was complexed with DS before NP formation, providing peptide loading and protease protection. **e)** Rheology confirmed suitability for topical cream application. **B)** Physicochemical characterization. **a)** Colloidal aggregates formed upon mixing pNPs and nNPs. **b)** ZP analysis showed slightly negative charge. **c)** Hydrodynamic sizes of pNPs and nNPs were in the nm range, while pNP:nNP aggregates reached μm–mm sizes. **C)***In vitro* antibacterial activity. **a)** Workflow of protease-challenged assays. **b)** PA-13-loaded nano-network retained activity after trypsin exposure, reducing *P. aeruginosa* counts by ∼5 log_10_ CFU mL^−1^ compared with free PA-13. **D)***Ex vivo* porcine skin infection model. **c)** Workflow of skin infection and treatments. **d)** Images of infected skin treated with PA-13-loaded network vs controls over 24 h. **e)** Bacterial reductions at 24 h. Values were log_10_ transformed and presented as the mean + SD (*N* = 6, *n* = 1). *p*-values were determined using a two-way ANOVA with Tukey's post hoc test. Significant differences are indicated in GP style: >0.05 (ns), ≤0.05 (∗), ≤0.01 (∗∗), ≤0.001 (∗∗∗), ≤0.0001 (∗∗∗∗). Reproduced with permission [197]. Copyright 2022, Wiley.

Considering that susceptibility to enzymatic degradation and low bioavailability of AMPs reduce its clinical translation, Klubthawee et al. [197] reported a colloidal nano-network assembled from chitosan (CS) and dextran sulfate (DS), where the antimicrobial peptide PA-13 was initially complexed with DS before incorporation into the nanosystem, ensuring stability and efficient loading (Fig. 6A). The resulting aggregates exhibited a net negative charge (−20 mV) and reached micro-to millimeter dimensions, as confirmed by cryo-scanning electron microscopy (Cryo-SEM, Fig. 6B). This design provided marked protection against enzymatic degradation. In trypsin assays, free PA-13 rapidly lost activity, whereas the nano-network retained antibacterial potency, reducing *P. aeruginosa* ATCC 27853 counts by nearly 5 log_10_ CFU mL^−1^ (Fig. 6C). *Ex vivo* experiments using porcine skin infected with *P. aeruginosa* corroborated these results, achieving ∼2.7 log_10_ CFU reduction at 24 h compared with free PA-13, the unloaded network, or a commercial cream, while histological analysis showed preserved epidermal and dermal architecture after treatment (Fig. 6D).

These findings demonstrate how polymeric nano-networks can safeguard AMPs from proteolysis and sustain topical efficacy in infection models. Unlike lipid-based carriers that rely on bilayer packing or crystallinity, the polyelectrolyte interactions and gel-like organization of polymeric systems open complementary strategies for AMP stabilization and localized delivery against Gram-negative pathogens.

Following these advances with colloidal nano-networks, more refined designs have focused on dendrimer–polymer conjugates that offer additional control over charge distribution and peptide orientation, Jordan et al. [198] developed antimicrobial peptide dendrimers (AMPDs) covalently conjugated to functionalized chitosan derivatives, aiming to reduce the intrinsic toxicity of dendrimers while maintaining their antibacterial potency against *P. aeruginosa*. By introducing cysteine residues to the dendrimers prior to conjugation, stable thioether linkages were established, which redirected highly cationic scaffolds into polymeric frameworks with improved charge modulation. The conjugates achieved lower MIC values compared with their free counterparts and exhibited reduced hemolysis as well as negligible fibroblast toxicity, confirming that this strategy improved both selectivity and safety. What distinguishes this approach is that the covalent linkage modulates both the spatial orientation and charge distribution of AMPDs, improving selectivity toward bacterial membranes while limiting nonspecific damage to mammalian cells. This demonstrates that polymer–peptide conjugates act not merely as carriers but as active modifiers of peptide behavior, expanding the therapeutic window of molecules previously constrained by toxicity.

Representative organic nanoplatforms (lipidic, polymeric, dendrimeric, and hybrid systems) reported over the last five years, together with key *in vivo* outcomes, are summarized in Table 3.

**Table 3:** Summary of several organic nanomaterials for antimicrobial therapy.

Types of organic materials	Size/ZP (mV)	AMP	Bacteria tested	Highlights *in vitro*	Highlights *in vivo*	Ref.
FM-P/CS@OH-CATH30	164.6; −37.6	OH-CATH30	*E. coli*, *S. aureus.*	≥80 % kill at 3 mg mL^−1^; ↑ cell proliferation at 0.5 mg mL^−1^.	Rodent, wound: 0.27 % → wound closure 83.7 % (6 days), 98.5 % (12 days).	[199]
DEX@SET-M33	18; −13	SET-M33	*P. aeruginosa.*	>99.9 % kill ≥4 μg mL^−1^; ↓ cytotoxicity vs free.	Pulmonary, rodent: lung t½ 13 h (vs 1.21 h); better low-dose efficacy.	[200]
AA139-MCL/AA139-PNP	20; −2.1	AA139	*K. pneumoniae* (β-lactamase +).	Greater activity than the free peptide.	Rat, intratracheal: 0.25 mg → bactericidal at 24 h (MCL); 0.5 mg → ↑ survival; lung deposition ∼85 % (IT).	[201]
AB@LL-37	33.3; +5.7	LL-37	*P. aeruginosa.*	∼75 % kill (vs 60 % free).	Mouse, lung: ↓ TNF-α/IL-6, biofilm and lung damage.	[202]
Mast-CsNC	156; +54.9	Mastoparan	*A. baumannii* (MDR).	MIC_90_ 2 μg mL^−1^ (vs 16 free).	Rodent, reduces infection in lower concentrations.	[203]
D-TZP (polymyxin B)	247; −21	PMB	*K. pneumoniae.*	Slightly ↑ MIC vs free.	Mouse, CLP sepsis: MTD × 2; survival 75 % (vs 33 % free); ↓ inflammation/organ damage.	[204]
NN@PA-13	—; −20	PA-13	*P. aeruginosa.*	5 log_10_ CFU↓ under proteases.	Porcine skin (*ex vivo*): 2.7 log_10_ CFU↓ at 24 h.	[205]
LAOOH-OPA NPs	225–260; ∼+15	D-peptide	*S. aureus*, *E. coli.*	Broad-spectrum; minimal hemolysis/cytotoxicity.	Mouse, skin abscess: 1.4 log_10_↓ (*S. aureus*) vs vancomycin 0.9.	[206]
PMB-cubosomes	150–170; −23.8 to +1.6	PMB	*A. baumannii*, *P. aeruginosa*, *K. pneumoniae.*	Inhibition 97–98 %; *K. p.* 98.3 %.	Mouse, infection: 99–100 % burden reduction.	[207]
Colistin/Alg NPs	112; +0.7	Colistin	*E. coli* (ETEC, resistant).	N.R.	Pigs: diarrhea 0 % (vs 30 % commercial); ↓ fecal *E. coli.*	[208]
C16-A3K4(DMA)-CONH_2_ NPs	34	C16-A3K4(DMA)-CONH_2_	*S. cerevisiae*, *S. aureus*, *E. coli*, MRSA.	IC_50_ 41.7 μg mL^−1^ (*S. cerevisiae*); *S. aureus* 3.22 log_10_↓ 50–200 μg mL^−1^; *E. coli* 63 %↓ 100 μg mL^−1^.	Rodent: no toxicity ≤300 mg kg^−1^.	[209]
MelNP	>1000; −50 to +50	Melittin	*E. coli*, *P. aeruginosa*, *S. aureus.*	MIC 2–4 μM, MBC 2–8 μM; hemolysis 54.9 % at 10 μM.	Zebrafish embryos: LC_50_ 6.13 μM.	[210]
ConA-CS NPsatCM11	350; +22.3	CM11	*H. pylori.*	MIC (peptide) 16 μg mL^−1^; 32 μg mL^−1^ (NPs) → complete kill at 24 h; no cytotoxicity.	Reduces gastric inflammation; IL-1β ↓; matches triple therapy.	[211]
PMB/ML-niosomes	131–228.6; −17 to −48.5	Polymyxin B.	*K. pneumoniae*, *A. baumannii*, *P. aeruginosa.*	Potent, synergistic.	Lung & kidney burden ↓; outperforms PMB; low toxicity.	[212]
TA-dendrimers	N.R.	Triazine dendrimeric peptides (Gen 1–2).	*E. coli*, *P. aeruginosa.*	Time-kill <1 h at MIC; no hemolysis ≤1 mg mL^−1^; >70 % viability ≤64 μg mL^−1^.	Mouse (*H. pylori* model): chitosan NPs → 75 % bacterial load ↓ [14d].	[213]
Endolysin@Ag–CBS dendrimer	350–800	Lysin.	*P. aeruginosa.*	MIC 64–128 μg mL^−1^; ROS ↑.	Zebrafish embryos: low toxicity ≤0.5 μM; ≥1 μM → malformations/mortality.	[214]
SPRAY-BLKR NPs	20	BLKR.	*E. coli*, MRSA.	Lung-deposited; membrane disruption; overcomes MDR.	Mouse, ALI: >98 % clearance; ∼73 % survival; high pulmonary retention; no systemic toxicity.	[215]
FFN NPs	69.8–70.7; +21.3 to +24	N6-derived peptide (FFN).	*E. coli*, *S. aureus.*	MIC 2.52 μM; post-antibiotic effect ≤5.3 h; hemolysis 6.08 %.	Mouse: well-tolerated ≤10 mg kg^−1^ (no toxicity).	[216]

∗Polyvinyl alcohol/chitosan electrospun fiber membrane loaded with OH-CATH30 nanoparticles (FM-P/CS@OH-CATH30); dextran nanoparticles loaded with SET-M33 (DEX@SET-M33); lipid-core micelles loaded with AA139 (AA139-PNP); polymeric nanoparticles loaded with AA139 (AA139-MCL); albumin-based LL-37 peptide nanoparticles (AB@LL-37); mastoparan–chitosan nanoconstructs (Mast-CsNC); vitamin D-loaded tannic acid/iron nanocapsules coated with low-molecular-weight zwitterionic chitosan and polymyxin B (D-TZP); PA-13 loaded nano-network (NN@PA-13); linoleic acid hydroperoxide–D(KLAK)_2_ self-assembled nanoparticles (LAOOH-OPA NPs); polymyxin B-loaded cubosomes (PMB-cubosomes); colistin-loaded alginate nanoparticles (Colistin/Alg NPs); C16-A3K4(DMA)-CONH_2_ nanoparticles (C16-A3K4(DMA)-CONH_2_ NPs); self-assembled melittin nanoparticles (MelNPs); concanavalin A–coated chitosan nanoparticles loaded with CM11 peptide (ConA-CS NPs@CM11); polymyxin B/monolaurin-based niosomes (PMB/ML-niosomes); 1,3,5-triazine-based antimicrobial peptide dendrimers (TA-dendrimers); endolysin CHAP domain–carbosilane silver metallodendrimer complex (Endolysin@Ag–CBS dendrimer); SPRAY-BLKR nanoparticles (SPRAY-BLKR NPs); self-assembling FFN antimicrobial peptide nanoparticles (FFN NPs); minimum inhibitory concentration (MIC); minimum bactericidal concentration (MBC); lethal concentration for 50 % of organisms (LC_50_); zeta potential (ZP); half-life (t½); post-antibiotic effect (PAE); not reported (N.R.); reactive oxygen species (ROS); acute lung injury (ALI); colony-forming units (CFU); maximum tolerated dose (MTD); multidrug-resistant (MDR); enterotoxigenic *Escherichia coli* (ETEC); cecal ligation and puncture (CLP); intratracheal (IT); methicillin-resistant *Staphylococcus aureus* (MRSA); tumor necrosis factor alpha (TNF-α); interleukin-6 (IL-6); interleukin-1 beta (IL-1β). Symbols are also employed for conciseness: ↑ (increase), ↓ (decrease), and → (leads to/results in).

While polymeric nanoparticles offer tunable degradation and functionalization, they may be insufficient when photo- or magneto-activable responses or higher physicochemical robustness are required. In such cases, inorganic cores provide complementary mechanisms—such as photocatalysis, reactive oxygen species (ROS) generation, or magnetic guidance—that can be further enhanced through peptide coupling [217].

### 4.3 Inorganic nanoparticles

Inorganic nanoparticles have attracted significant attention from researchers due to their structural diversity, tunable properties, and exceptional functional versatility [218,219]. Among the various existing classes, metallic nanoparticles (MNPs), mesoporous silica nanoparticles (MSNs), and metal oxide nanoparticles (MONs) stand out for their unique physicochemical properties and broad range of applications [220,221].

#### 4.3.1 Metallic nanoparticles

MNPs feature conductive cores that support surface plasmon resonance (in Au/Ag) and allow controlled ion release (Ag^+^/Cu^2+^) that enhances antibacterial activity [222]. Their surface chemistry enables covalent and coordinative anchoring (Au–S linkages with thiols; interactions with amines/imidazoles), permitting oriented, high-density display of antimicrobial peptides and enabling photothermal effects, surface-enhanced Raman scattering, and other photophysical responses not accessible with oxides [223,224]. MNPs also possess robust resistance to corrosion and oxidation. Nevertheless, while several nanosystems demonstrate acceptable biocompatibility when appropriately coated and dosed, concerns remain regarding dose- and surface-dependent cytotoxicity as well as potential environmental impact [225]. They are also capable of generating reactive oxygen species (ROS) and exerting photothermal and gene-silencing effects, further expanding their potential in targeted therapies [226]. MNPs can be synthesized using physical, chemical, or biological methods, following two main approaches: top-down and bottom-up. The top-down approach reduces bulk materials to the nanoscale using techniques such as laser ablation, mechanical milling, lithography, or thermal decomposition [227]. In contrast, the bottom-up approach builds nanoparticles from atoms or molecules through processes such as sol-gel, molecular self-assembly, chemical reduction, or biosynthesis. Chemical methods typically use reducing and stabilizing agents — such as sodium borohydride or citrate — to control particle size and prevent agglomeration, although they often involve toxic reagents [228]. Physical methods require extreme temperature and pressure conditions, increasing production costs. On the other hand, biological synthesis employs plant extracts or microorganisms such as bacteria and fungi, offering a greener and more economical alternative, albeit with limitations in morphological control [229]. These synthesis strategies enable surface modification with polymers, biomolecules, or functional ligands, adapting the nanoparticles for specific applications. Nevertheless, challenges such as aggregation, size uniformity, cytotoxicity, and environmental impact persist and must be carefully considered in their development and application [230].

**Fig. 7:**
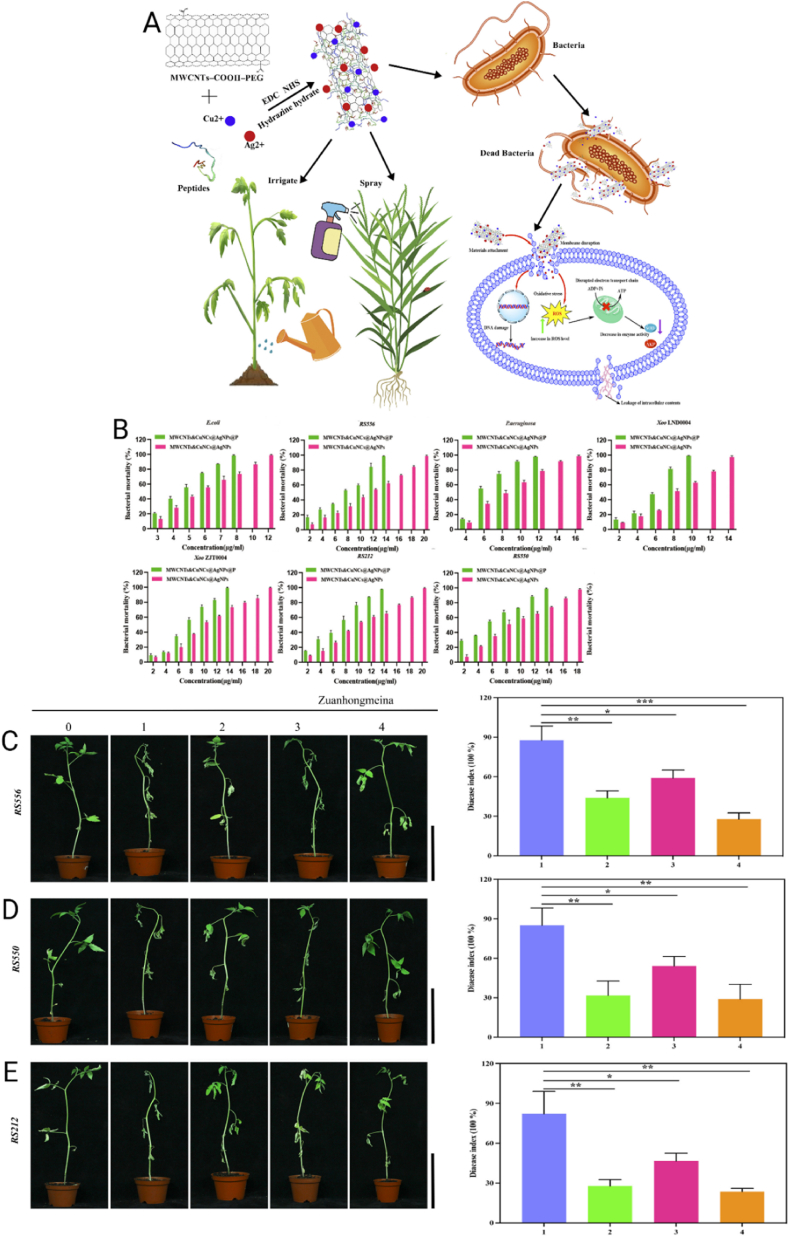
**A**) Schematic representation of the MWCNTs&CuNCs@AgNPs@P and its antibacterial mechanism. **B**) Bacterial mortality of *E. coli*, RS556, *P. aeruginosa*, *Xoo* LND0004, *Xoo* ZJT0004, RS212, and RS550 after incubation with MWCNTs&CuNCs@AgNPs@P and MWCNTs&CuNCs@AgNPs at different concentrations. Data are presented as the mean ± SD, n = 3. **C-E**) Glasshouse experiments with bacterial wilt. Antibacterial activity of the MWCNTs&CuNCs@AgNPs@P and MWCNTs&CuNCs@AgNPs as a curative technique against *R. solanacearum* in tomato plants in pot culture trials. **0)** healthy tomato, **1)** infected control, **2)** after spraying with 20 μg mL^−1^ of MWCNTs&CuNCs@AgNPs@P, **3)** after spraying with 20 μg mL^−1^ of MWCNTs&CuNCs@AgNPs, **4)** after spraying with 20 % thiodiazole-copper (diluted 500-fold). All plants were tested in a randomized complete block design. The scale bar represents 20 cm. Data are shown as the mean ± SD; n = 3. Reproduced with permission [231]. Copyright 2024, Elsevier.

The growing use of chemical pesticides with low effectiveness and absence of reliable eco-friendly bactericides have improved the persistent pathogenic bacteria in crops, impacting food production security besides promoting high losses of global crop yields. To address these issues, Ning et al. [231] engineered a multifunctional nanocomposite that integrated a short synthetic AMP with copper nanoclusters (CuNCs) and silver nanoparticles (AgNPs) anchored onto polyethylene glycol–modified multiwalled carbon nanotubes (MWCNTs, Fig. 7A). The design aimed to exploit the intrinsic antimicrobial properties of silver and copper while lowering the required metal dose through synergistic interaction with the peptide. Structural analysis confirmed the stable anchoring of the metallic nanoparticles on the nanotubes, with a reduction in average size after peptide conjugation, indicating that the peptide influenced assembly. Antibacterial testing demonstrated that the peptide–MWCNT–Cu/Ag nanoplatform was more effective than non-peptide controls, achieving MIC values of 8 μg mL^−1^ against *E. coli*, 16 μg mL^−1^ against *P. aeruginosa*, and intermediate values for other Gram-negative plant pathogens (Fig. 7B). In a tomato irrigation model challenged with *Ralstonia solanacearum*, the nanoformulation outperformed both conventional copper-based pesticides and the non-peptide nanocomposite, markedly reducing bacterial burden and protecting plants from wilt disease (Fig. 7C–E). Importantly, the amount of copper and silver used was significantly lower than in commercial agrochemicals, reducing the potential environmental load.

The study illustrates how the combination of AMPs with metallic nanostructures can reshape their functional profile. Carbon nanotubes increase the available surface area and provide a scaffold for multi-component integration, while metallic species contribute ion-mediated toxicity and AMPs enhance selectivity through membrane disruption. This cooperative mechanism improves efficacy and addresses the challenge posed by high metal dosages, which often limit the sustainability of nanoparticle-based bactericides. The broader implication is that hybrid nanosystems can extend the application of AMPs beyond the clinical domain into agriculture, where plant pathogens threaten global food production. At the same time, advancing this strategy requires systematic evaluation of environmental safety, long-term stability, and scalable synthesis routes in order to balance efficacy with ecological responsibility.

#### 4.3.2 Mesoporous silica nanoparticles

MSNs stand out due to their large surface area, pore volume, and ability to enable high drug loading and controlled release of pharmaceuticals, proteins, or nucleic acids [232,233]. While silica materials are recognized by the FDA as generally recognized as safe, the biocompatibility of MSN at the nanometric scale is not absolute but depends strongly on particle size, surface chemistry, and dosage [234]. Their size and morphology are easily tunable (50–200 nm), and both internal and external surfaces can be easily functionalized, allowing the incorporation of therapeutic and imaging agents for simultaneous diagnostic and therapeutic applications [229,235]. MSNs offer controlled release capabilities for a wide variety of therapeutic and diagnostic payloads, significantly surpassing the loading capacity of many organic delivery systems [236]. MSNs are commonly synthesized through methods such as the Stöber process, based on sol-gel reactions that induce the condensation of precursors like Tetraethyl orthosilicate (TEOS) in the presence of surfactants such as cetyltrimethylammonium bromide (CTAB), which guide pore formation [237,238]. Another widely used method is evaporation-induced self-assembly, where solvent evaporation drives the organization of micellar structures that guide formation of the mesoporous framework [239]. Ultrasonication- and microwave-assisted synthesis methods are also employed, as they accelerate production, improve structural homogeneity, and reduce reaction times. While these approaches facilitate laboratory-scale preparation, achieving reproducible control in large-scale production remains a significant technical and economic challenge [240]. Despite their advantages, MSNs present critical limitations that must be addressed. One of the main drawbacks is the possible *in vivo* toxicity associated with organ accumulation at high doses, particularly if surface functionalization, particle size, or administration parameters are not properly controlled, which may trigger inflammatory responses in organs such as the liver and spleen [241]. The typical negative surface charge of silica can limit the EE of negatively charged molecules, and its rigid structure can hinder controlled drug release unless properly modified. Furthermore, large-scale production with reproducible control over size and functionalization still represents a significant technical and economic challenge [229].

**Fig. 8:**
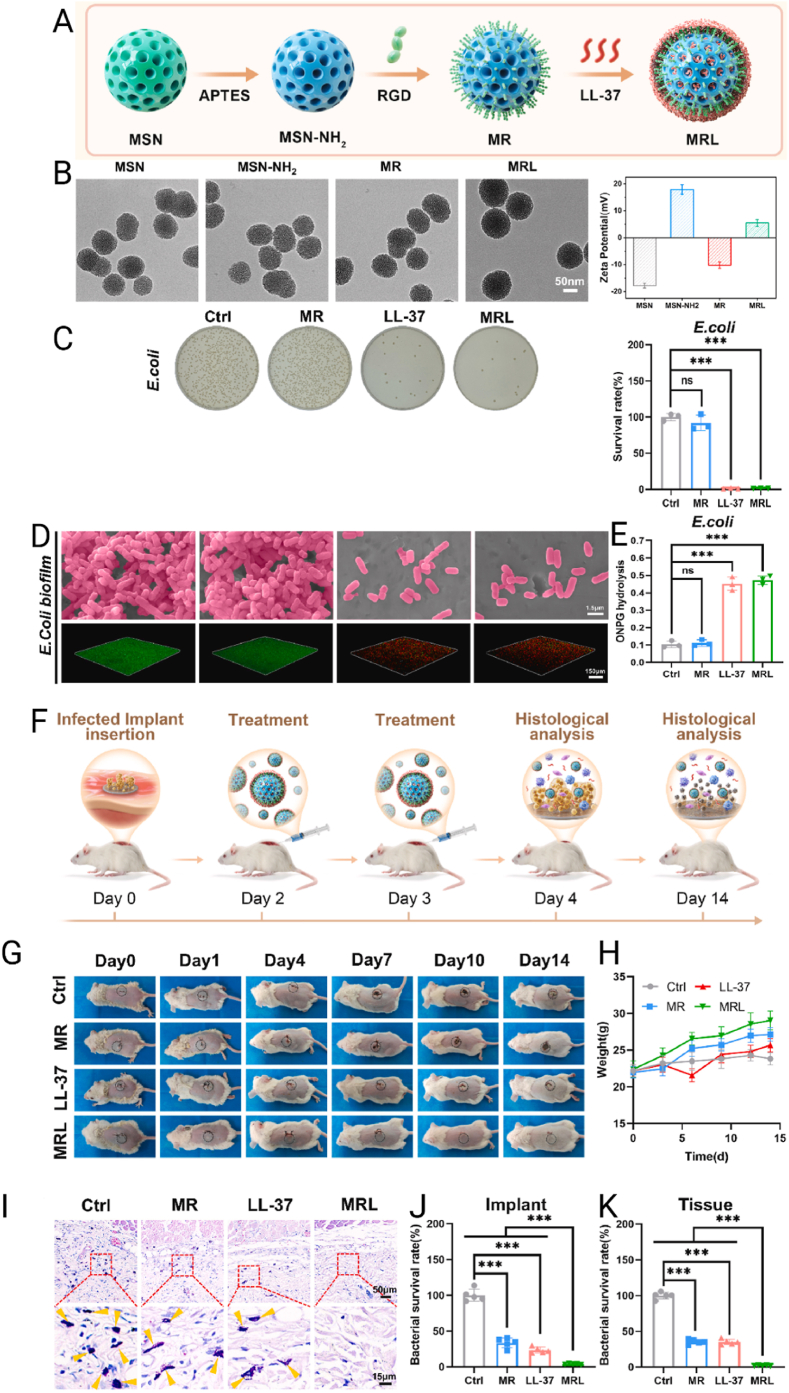
**A)** Schematic of MSN synthesis, RGD conjugation (MR), and LL-37 loading (MRL). **B)** TEM images and ZPs of MSN, MSN-NH_2_, MR, and MRL (scale bar: 100 μm). **C)** Antibacterial activity against *E. coli* shown by scanning probe microscopy (SPM) images and quantitative analysis (n = 3). **D)** SEM and CLSM 3D images of *E. coli* biofilms after Syto9/PI staining (scale bars: 1.5 μm, 150 μm). **E)** Membrane permeability of *E. coli* measured by ONPG hydrolysis. ns = not significant; ∗p < 0.05, ∗∗p < 0.01, ∗∗∗p < 0.001. **F–G)***In vivo* subcutaneous implant infection model and representative macroscopic images of mice post-treatment. **H)** Changes in body weight over 14 days (n = 5). **I)** Giemsa-stained tissue sections around implants on day 14 (scale bars: 1.5 μm, 150 μm). **J–K)** Bacterial survival on implants and surrounding tissues (n = 5). The data are presented as the means ± SD, p < 0.05, ∗∗p < 0.01, and ∗∗∗p < 0.001. Reproduced with permission [242]. Copyright 2025, Elsevier.

Aiming to avoid implant-related infections by biofilms or MDR bacteria strains, Z. Li et al. [242] developed MSNs functionalized with the tripeptide arginine–glycine–aspartate (RGD) and loaded with LL-37 with the aim of counteracting implant-associated infections. Progressive surface modifications confirmed the generation of a stable nanosystem with physicochemical features suitable for peptide release (Fig. 8A and B). Antibacterial assays showed that the loaded nanoformulation (MRL) maintained equivalent efficacy to free LL-37 against *E. coli*, accompanied by clear biofilm disruption in confocal and electron microscopy analyses (Fig. 8C–E). Beyond antimicrobial action, a distinctive outcome was the ability of RGD to reduce macrophage pyroptosis, thereby improving viability and sustaining immune activity. This immunomodulatory effect preserved host defense while maintaining antibacterial efficacy. In a murine subcutaneous implant infection model, MRL outperformed controls by promoting superior healing and recovery, as well as near-complete elimination of bacterial burden in implants and surrounding tissues (Fig. 8F–K). These findings highlight that MSNs can extend beyond their role as passive nanocarriers for controlled drug release. Incorporating a bioactive ligand such as RGD demonstrates that antimicrobial action, biofilm disruption, and immunomodulation can be integrated into a single multifunctional nanocarrier. The most relevant contribution lies in showing that MSN surfaces can directly modulate immune cell interactions, mitigating detrimental inflammatory responses that otherwise limit the therapeutic use of peptides such as LL-37. This approach redefines the application of MSNs in biomaterial-associated infections, shifting from merely pathogen eradication toward the promotion of a tissue-regenerative microenvironment. Nevertheless, clinical translation will require confirmation of the stability of the immunomodulatory effect under more complex physiological conditions, reduction of long-term organ accumulation, and scalable synthesis protocols with reproducible control of particle size and surface functionalization.

**Fig. 9:**
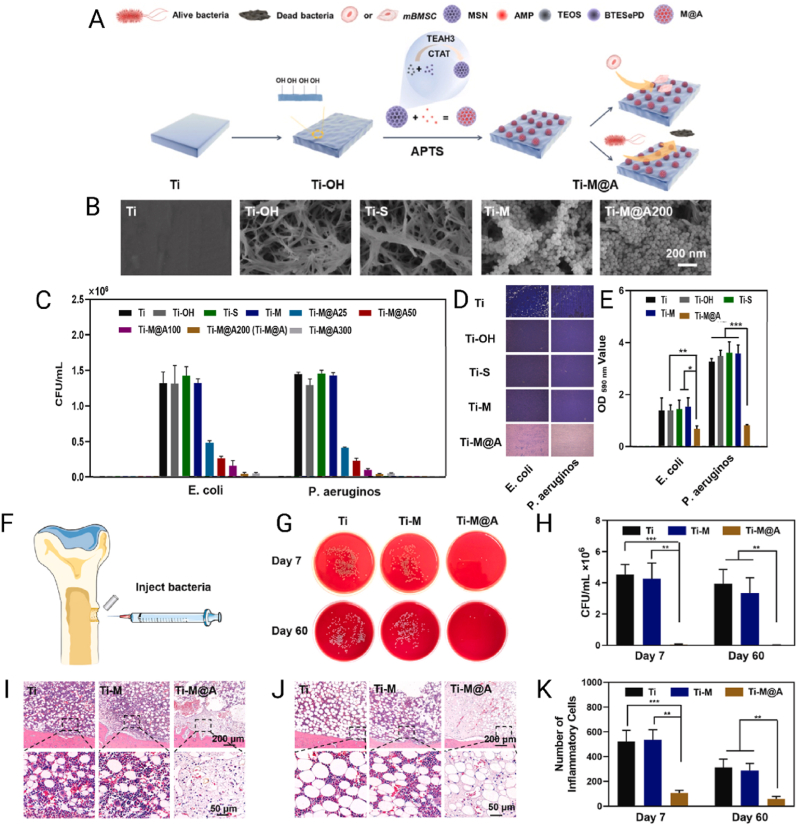
**A**) Schematic diagram showing the preparation process of the MSNs (labeled M), MSNs@HHC36 (labeled M@A) and functionalized titanium implants (labeled Ti-M@A and Ti-M). **B**) FE-SEM images of different functionalized Ti surfaces. *In vitro* antimicrobial activities of the functionalized titanium surfaces against **C**) *E. coli* and *P. aeruginosa* determined by the agar plate method (n = 3). **D**) Biofilm crystal violet staining of the indicated bacteria on the functionalized surfaces. **E**) Quantitative analysis of biofilm crystal violet staining (n = 3) ∗ denotes p < 0.05, ∗∗ denotes p < 0.01, ∗∗∗ denotes p < 0.001. *In vivo* assay in a bone defect/infection model: **F**) The surgical procedure of the *in vivo* rabbit animal model, **G**) The bacterial culture results of the indicated implants after 7 and 60 days of implantation, **H**) Antimicrobial activity of the indicated implants after 7 and 60 days of implantation. The H&E staining images of the tissues around the implants after **I**) 7 and **J**) 60 days of implantation. The high-magnification images were taken from the black rectangles. **K**) The quantification of inflammatory cells calculated from the H&E images after 7 and 60 days of implantation (n = 3). Reproduced with permission [243]. Copyright 2025, Elsevier.

One of the main issues that compromises orthopedic implantations is the development of bacterial infection on the implanted site causing also slow osseointegration. In this sense, Dong et al. [243] developed titanium-based implants functionalized with diselenide-bridged MSNs loaded with the antimicrobial peptide HHC36 (M@A) to prevent infection and inflammation in orthopedic applications. Surface activation of titanium and subsequent conjugation produced stable coatings (Fig. 9A and B). *In vitro* assays demonstrated that Ti-M@A reduced the viability of *E. coli* and *P. aeruginosa* by more than 95 % in a dose-dependent manner (Fig. 9C), along with partial inhibition of biofilm formation (Fig. 9D and E). In a rabbit femoral infection model, coated implants nearly eradicated bacterial load after 7 and 60 days (Fig. 9F–H). Histological evaluation revealed lower inflammatory infiltration and improved tissue compatibility in Ti-M@A implants compared with controls (Fig. 9I–K). The most relevant outcome is that the coating transforms the implant into an active interface capable of responding to infectious microenvironments while modulating processes that typically compromise device survival. The diselenide bridges enabled oxidative condition–responsive release, whereas HHC36 maintained sustained antimicrobial action. The practical consequence was a simultaneous reduction in bacterial colonization and in the inflammation that interferes with bone regeneration. This integration is particularly critical in orthopedics, where infection and impaired osseointegration are interdependent processes that often lead to implant failure. From a broader perspective, this strategy highlights how nanocoatings can be designed as multifunctional platforms that regulate interactions not only with pathogens but also with host tissues, thereby aligning infection control with regenerative outcomes. The long-term validation in animal models strengthens the translational potential of this approach, especially if the response of the coating is further optimized for patients with high-risk conditions such as diabetes or osteoporosis.

#### 4.3.3 Metal oxide nanoparticles

Among inorganic nanomaterials, MONs such as TiO_2_, ZnO, CuO, and Fe_3_O_4_ represent a distinct class of semiconductors with tunable band gaps. Their nanoscale dimensions endow them with size- and surface-dependent properties, including photocatalytic activity and light- or pH-triggered generation of ROS, as well as magnetic responsiveness in the case of Fe_3_O_4_ [244]. These features underpin applications that span environmental remediation, advanced imaging, targeted drug delivery, and controlled release of active compounds [230,245,246]. MONs also exhibit high chemical and thermal stability and facile surface functionalization, where amphoteric hydroxyl groups enable electrostatic interactions and silane coupling for peptide conjugation [247,248].

Their synthesis can be achieved through chemical methods (such as precipitation or sol-gel), hydrothermal processes, and green synthesis routes. The latter employ plant extracts, microorganisms, or enzymes as reducing or stabilizing agents to improve biocompatibility and reduce environmental impact [249,250]. Despite their many advantages, metal oxide nanoparticles face limitations such as low solubility, potential toxicity, toxic residues from chemical synthesis, tendency to aggregate in biological media, and low delivery efficiency due to rapid uptake by non-target organs. Moreover, if not properly functionalized, they can induce immune responses inflammation or hypersensitivity [[251], [252], [253]].

One of the most promising trends in the field of inorganic nanoparticles is their integration with peptide-based systems to create multifunctional nanoplatforms aimed at targeted therapy, antimicrobial applications, and controlled drug release [11]. Peptides provide a bioactive interface that enhances specificity, cellular uptake, and the therapeutic efficacy of nanoparticles [254,255]. The conjugation or encapsulation of antimicrobial peptides within inorganic nanoparticles not only protects them from enzymatic degradation but also leverages the physicochemical advantages of the inorganic core, resulting in enhanced antimicrobial potency and selectivity while minimizing toxicity to healthy tissues [[256], [257], [258]]. Representative AMP–inorganic nanoplatforms from the last five years, including physicochemical features, antimicrobial targets, and *in vivo* results, are summarized in Table 4.

**Table 4:** Summary of several inorganic nanomaterials for antimicrobial therapy.

Type of inorganic materials	Size/ZP (mV)	AMP	Bacteria tested	Highlights *in vitro*	Highlights *in vivo*	Ref.
HA-P(Au/Ag)	128; −9.57	Scolopendrasin IX	MDR *A. baumannii*	Hemolysis <2 % viability >90 %; MIC ↓ 64 % (16 → 4 μg mL^−1^).	Mouse with NIR survival ↑ 80 % colonies ↓ 95 % lung health ↑.	[260]
AP-CdSe	9–14	Periplanetasin-4	*E. coli*, *S. aureus*	MIC ↓ to 4–24 μg mL^−1^; 40 μg mL^−1^ → viability 3.8–10.3 % apoptosis ↑ 98.8–99.1 %.	Mouse *S. aureus* bacteremia survival ↑ 83 %.	[261]
AgNPs-AMP@PSiMPs	14.2	Tet-213	*E. coli*, *S. aureus*	MIC 1.5 mg mL^−1^ (*E. coli*) 2 mg mL^−1^ (*S. aureus*); cytotoxicity <10 %.	Rat infected wounds injured area ↓ and colonies ↓ 3 d.	[262]
MSN@GA/CO	∼190; −21.6	Colistin	*E. coli*	MIC/MBC 1.953/3.906 μg mL^−1^ MBIC/MBEC 1.953/7.813 μg mL^−1^; low cytotoxicity.	Infected bone model biofilm ↓ 99.9 % at 62.5 μg mL^−1^.	[263]
AuNP-AptHis	N.R	Lys AB2 P3His	*A. baumannii*	MIC 2–4 μM; intracellular bacteria ↓ 50 %.	Mouse survival ↑70 % spleen load ↓ 3.5 ×	[264]
TTCT	∼25; −14.99	S-thanatin	TRKP (*K. pneumoniae*)	MIC 1 μg mL^−1^.	Mouse lungs nanorods accumulation biodistribution ↑.	[265]
AgNPs@AMP	425; ∼+25	(LLRR)_3_	*E. coli*, *S. aureus*	MIC 2.5 (*E. coli*)/1.25 μg mL^−1^ (*S. aureus*).	Mouse wound healing ↑ vs AMP and AgNPs.	[266]
Ti-MSN@AMP	50; −12.1	HHC36	*E. coli*, *P. aeruginosa*, MRSA, *S. aureus*	broad-spectrum reduction 95.7–98.5 %.	*S. aureus* reduction 98.82 % (7 d), in rabbit bone defect model.	[267]
Col-Ag-CuO	190.25	Colistin	*P. aeruginosa*	MIC 0.0117 μg mL^−1^; swarming ↓ 32 %/64 %, twitching ↓ 34 %/97 % (two doses).	*Galleria mellonella* survival 100 % (3 days); virulence ↓.	[268]

∗Hybrid gold/silver nanocages modified with hyaluronic acid and an antimicrobial peptide (HA-P(Au/Ag)); antimicrobial-peptide-functionalized cadmium selenide quantum-dot fluorescent nanoparticles (AP-CdSe); porous silicon nanocarriers co-loaded with silver nanoparticles and an antimicrobial peptide (AgNPs-AMP@PSiMPs); mesoporous silica nanoparticles coated with gum arabic/colistin (MSN@GA/CO); gold nanoparticles bearing a DNA aptamer and loaded with an antimicrobial peptide (AuNP-AptHis); TPGS-based S-thanatin-functionalized nanorods (TTCT); antimicrobial peptide–conjugated silver nanoparticles (AgNPs@AMP); titanium implants functionalized with mesoporous silica nanoparticles and loaded with an antimicrobial peptide (Ti-MSN@AMP); silver–copper oxide nanoparticles loaded with colistin (Col-Ag-CuO); tigecycline-resistant *Klebsiella pneumoniae* (TRKP); minimum inhibitory concentration (MIC); minimum bactericidal concentration (MBC); minimum biofilm inhibitory concentration (MBIC); minimum biofilm eradication concentration (MBEC); near-infrared radiation (NIR); multidrug-resistant (MDR); methicillin-resistant *Staphylococcus aureus* (MRSA); zeta potential (ZP). Symbols: ↑ increase; ↓ decrease; → leads to/results in.

**Fig. 10:**
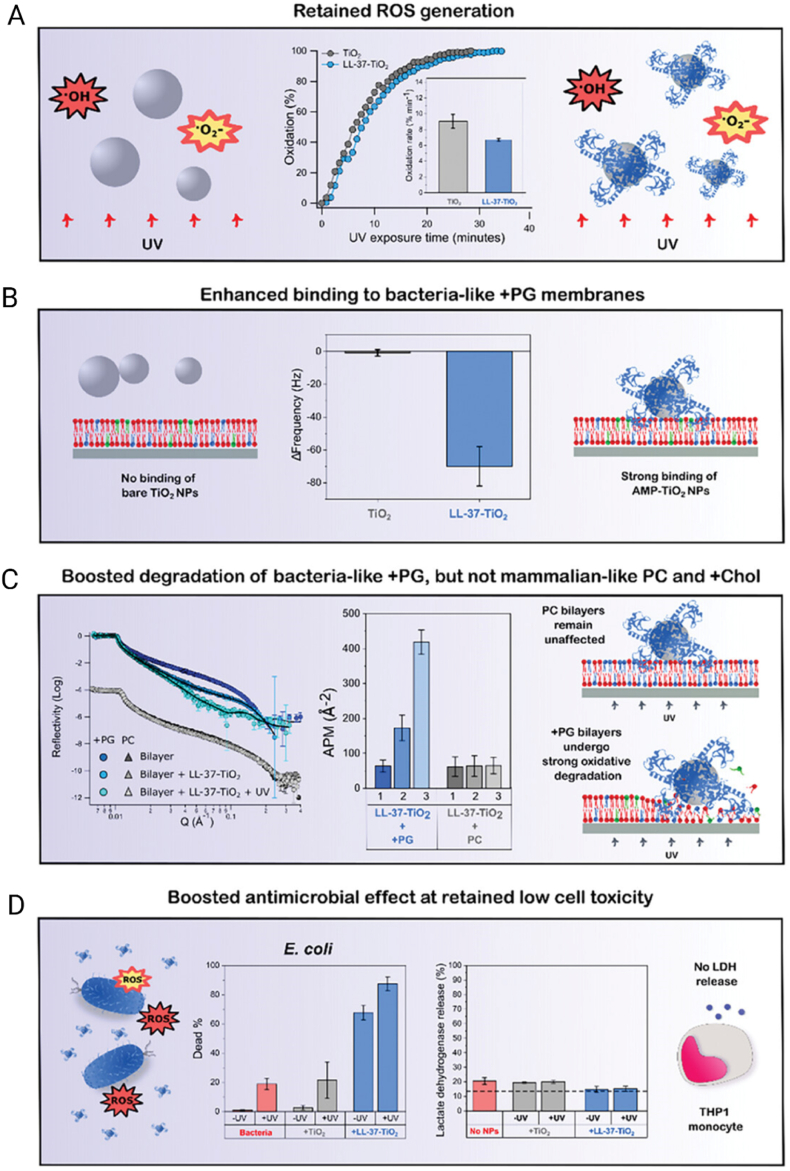
Schematic illustration of experimental results demonstrating the feasibility of coating photocatalytic NPs with AMPs for selective boosting of antimicrobial effects. **A)** On coating TiO_2_ NPs by positively charged LL-37 at pH 5.4–7.4, the peptide coating does not detrimentally interfere with ROS generation and displays good stability on UV exposure for 1–2 h. **B)** As a result of this, binding of peptide-coated TiO_2_ NPs is much higher than that of bare TiO_2_ NPs, particularly at anionic bacteria-like membranes, and **C)** oxidative degradation of the latter on UV exposure strongly boosted by peptide coating, whereas corresponding effects on zwitterionic mammalian-like membranes were much smaller. **D)** LL-37-coated TiO_2_ NPs displayed boosted antimicrobial effects against Gram-negative *E. coli*, whereas toxicity against human THP1 monocytes remained low. Reproduced with permission [259]. Copyright 2024, Wiley.

The substantial rise of resistant bacteria in recent years has driven the development of more effective antimicrobial systems. Among these, titanium dioxide nanoparticles (TiO_2_ NPs) stand out as photocatalytic nanomaterials capable of generating reactive oxygen species (ROS) under ultraviolet (UV) light, disrupting bacterial membranes and causing cell death. However, TiO_2_ NPs lack selective targeting of bacterial membranes, which can result in adverse effects on healthy cells due to indiscriminate ROS production. In this sense, Caselli et al. [259], demonstrated that titanium dioxide nanoparticles (TiO_2_ NPs), traditionally recognized for their photocatalytic capacity to generate reactive ROS under UV irradiation, can be transformed into selective antimicrobial agents through coating with antimicrobial peptides. A major limitation of bare TiO_2_ is the indiscriminate ROS production, which poses a risk of collateral damage to mammalian cells. Conjugation with LL-37 conferred directional effects toward bacterial membranes, thereby reorienting the photocatalytic activity. Biophysical analyses supported this selectivity. Although both bare TiO_2_ and LL-37–TiO_2_ produced comparable ROS levels upon UV exposure (Fig. 10A), only the functionalized nanoplatform showed strong affinity for anionic bacterial bilayers and induced their disruption, while mammalian-like bilayers remained unaffected (Fig. 10B and C). In biological assays, bare TiO_2_ exhibited negligible activity against *E. coli*, whereas LL-37–TiO_2_ eliminated 68 ± 5 % of bacteria in dark conditions and 88 ± 5 % following irradiation. Cytotoxicity assays further confirmed biocompatibility, with no lactate dehydrogenase release observed in human monocytes (Fig. 10D). These findings show that the peptide coating acts as more than a superficial modification; it functions as a molecular filter that converts an unspecific photocatalyst into a dual nanoplatform. The peptide drives selective targeting of bacterial membranes, while the inorganic core sustains ROS generation, creating a cooperative mechanism that enhances antimicrobial potency while sparing host cells. This approach redefines the role of TiO_2_-based nanomaterials, positioning them as precision nanotools in infectious contexts where membrane disruption and localized activation are decisive. The broader implication is that peptide–inorganic hybrids can provide a blueprint for designing nanosystems that merge catalytic activity with biological specificity, offering a pathway toward clinical translation if stability and performance are validated under physiologically relevant light conditions.

## 5 Conclusions and future perspectives

Gram-negative bacterial infections remain a major clinical challenge due to the outer-membrane barrier, efflux pumps, enzymatic degradation, and horizontal gene transfer that limit antibiotic efficacy. AMPs offer broad activity, rapid killing, and immunomodulation, yet translation is constrained by proteolytic instability, dose-related cytotoxicity, and production costs. Nanocarriers mitigate these barriers by shielding AMPs from degradation, enabling controlled release, improving targeting and intracellular accumulation, and thereby lowering effective doses while reducing systemic burden. Evidence across lipid, polymeric, and inorganic systems indicates MIC reductions and activity against biofilm- and efflux-mediated tolerance, but most AMP–nanocarrier programs remain preclinical; standardized characterization, long-term safety, and testing in clinically relevant models are still needed. Looking ahead, feasibility depends on good manufacturing practice-compatible unit operations and robust control of critical quality attributes (size, PDI, ZP, loading, release) under quality-by-design and stability programs, ideally supported by process analytical technology. Cost-effectiveness should be judged as cost per effective dose delivered to the target site and can be improved through shorter or modular peptides, high encapsulation efficiency, continuous manufacturing with solvent recovery, and room-temperature-stable presentations that ease logistics. Scalability favors scale-agnostic physics and platforms built on pharmacopeial excipients with validated analytics and early regulatory dialogue to streamline impurity profiling and bridging toxicology. Therapeutic impact can be further enhanced by rational synergies: AMP membrane priming to potentiate rifamycins, fluoroquinolones, or β-lactams; pairing with efflux inhibitors or outer-membrane permeabilizers; co-formulation with matrix disruptors for biofilms; dual-peptide constructs coupling a homing motif with the active AMP; and stimuli-responsive hybrids that localize action. Near-term priorities include process maps with cost of goods estimates, quantitative synergy metrics under biofilm and intracellular conditions, engineering runs to de-risk scale-up, and translational pharmacokinetics/pharmacodynamics linking exposure to effect. Framing development around feasibility, cost, and scale—together with mechanism-matched synergies—positions peptide-enabled nanoplatforms as a realistic route to curb Gram-negative resistance.

## CRediT authorship contribution statement

**Christian S. Carnero Canales:** Writing – review & editing, Writing – original draft, Validation, Investigation, Conceptualization. **Jessica Ingrid Marquez Cazorla:** Writing – original draft, Visualization, Validation, Investigation. **Renzo Marianito Marquez Cazorla:** Writing – original draft, Visualization, Validation, Investigation. **Rafael Miguel Sábio:** Writing – review & editing, Visualization, Validation, Supervision. **Hélder A. Santos:** Writing – review & editing, Visualization, Validation, Supervision, Resources, Project administration, Funding acquisition, Conceptualization. **Fernando Rogério Pavan:** Writing – review & editing, Visualization, Validation, Supervision, Resources, Project administration, Funding acquisition, Conceptualization.

## Ethics approval and consent to participate

Not applicable. There are no clinical studies, animal experiments, or human subjects.

## Declaration of generative AI and AI-assisted technologies in the writing process

During the preparation of this work, the authors used ChatGPT and Curie to enhance the readability and language of the manuscript. After using these tools and services, the authors thoroughly reviewed and edited the content as needed and took full responsibility for the final version of the published article.

## Declaration of competing interest

Renzo Marianito Marquez Cazorla is affiliated with the Nanovida Research Center, Arequipa, Peru, which may be considered a potential conflict of interest. The remaining authors declare no conflicts of interest.

## Data Availability

No data was used for the research described in the article.
